# Heterologous mammalian Akt disrupts plasma membrane homeostasis by taking over TORC2 signaling in *Saccharomyces cerevisiae*

**DOI:** 10.1038/s41598-018-25717-w

**Published:** 2018-05-16

**Authors:** Isabel Rodríguez-Escudero, Teresa Fernández-Acero, Víctor J. Cid, María Molina

**Affiliations:** Departamento de Microbiología y Parasitología, Facultad de Farmacia, Universidad Complutense de Madrid and Instituto Ramón y Cajal de Investigaciones Sanitarias, Madrid, Spain

## Abstract

The Akt protein kinase is the main transducer of phosphatidylinositol-3,4,5-*tris*phosphate (PtdIns3,4,5P_3_) signaling in higher eukaryotes, controlling cell growth, motility, proliferation and survival. By co-expression of mammalian class I phosphatidylinositol 3-kinase (PI3K) and Akt in the *Saccharomyces cerevisiae* heterologous model, we previously described an inhibitory effect on yeast growth that relied on Akt kinase activity. Here we report that PI3K-Akt expression in yeast triggers the formation of large plasma membrane (PM) invaginations that were marked by actin patches, enriched in PtdIns4,5P_2_ and associated to abnormal intracellular cell wall deposits. These effects of Akt were mimicked by overproduction of the PtdIns4,5P_2_ effector Slm1, an adaptor of the Ypk1 and Ypk2 kinases in the TORC2 pathway. Although Slm1 was phosphorylated *in vivo* by Akt, TORC2-dependent Ypk1 activation did not occur. However, PI3K-activated Akt suppressed the lethality derived from inactivation of either TORC2 or Ypk protein kinases. Thus, heterologous co-expression of PI3K and Akt in yeast short-circuits PtdIns4,5P_2_- and TORC2-signaling at the level of the Slm-Ypk complex, overriding some of its functions. Our results underscore the importance of phosphoinositide-dependent kinases as key actors in the homeostasis and dynamics of the PM.

## Introduction

Eukaryotic organisms have evolved complex finely-regulated signaling pathways that ensure viability in a challenging environment by triggering appropriate cellular responses to regulate adaptation. Plasma membrane (PM) is the interface between the cell and the outer environment, and thus hosts a plethora of receptors that couple to cytoplasmic signal-transducing proteins, typically GTPases and protein kinases. The lipid composition of the PM inner leaflet is an essential actor in signal transduction events^1,2^. Thus, lipid homeostasis must be tightly regulated to allow proper signaling. Minor lipids such as sphingolipids and phosphoinositides, concentrated at distinct PM microdomains, are often involved in signaling as transducing molecules^3^. Among them, PtdIns4,5P_2_ is a universal spatial marker for the inner leaflet of the PM in eukaryotic cells. Thus, many signaling modules that assemble at the PM involve adaptor proteins that bear PtdIns4,5P_2_-binding motifs, such as stretches of basic residues, pleckstrin homology (PH) domains or BAR domains, for example^2,4^.

The budding yeast *S. cerevisiae*, a basic model in cell biology, has greatly contributed to our understanding of cell signaling. Numerous yeast signaling modules, as well as proteins involved in endocytic and exocytic processes, bear PtdIns4,5P_2_-binding domains that contribute to PM recognition^5,6^. In the latest years, accumulated evidence indicates that the PM is organized in mosaic-like microdomains, the most prominent being the Membrane Compartment containing Pma1 (MCP), the Membrane Compartment containing Can1 (MCC; coincident with a furrow-like structure named eisosome), and the Membrane Compartment containing Tor2 (MCT)^7,8^, but there are probably less conspicuous PM microdomains^9^. MCP compartments have been reported to be active sites of endo- and exocytosis^10^, whereas the MCC and MCT are involved in PtdIns4,5P_2_ homeostasis^11^. The invaginated PM furrows of MCCs are characteristically marked by BAR domain-containing proteins Pil1 and Lsp1^12,13^. The MCT includes the target of rapamycin complex 2 (TORC2), which is integrated by the Tor2 catalytic protein kinase subunit and accessory proteins Lst8, Bit61, Avo1, Avo2, and Avo3, and is conserved in higher cells^14^. Although many aspects of TORC2 function in yeast remain obscure, its role in the regulation of the actin cytoskeleton for the control of polarized growth and endocytosis has been well established^15–17^. TORC2 acts by phosphorylating the turn (TM) and hydrophobic (HM) motifs of the redundant Ypk1 and Ypk2 proteins^17,18^, members of the AGC kinase superfamily that have been reported to be functional homologues of mammalian serum- and glucocorticoid-inducible kinase (SGK)^19,20^. Like other AGC kinases, Ypks also require phosphorylation at their activation T-loop. The protein kinases responsible are redundant Pkh1 and Pkh2^21,22^, counterparts of higher eukaryotic phosphoinositide-dependent kinase (PDK1)^20^. PM recruitment of Pkh kinases seems to rely on binding to the Pil1 eisosome component, as well as to phosphoinositide and sphingolipid lipid species^17^. In turn, Ypk1 and Ypk2 use the Slm1 and Slm2 proteins, two redundant adaptors that bind PtdIns4,5P_2_ via their PH domains to facilitate proximity to their activator kinases^23,24^. The Pkh/TORC2-Slm-Ypk pathway responds to temperature stress and other challenges and contributes to maintenance of PM homeostasis. Indeed, known downstream phosphorylation targets of this pathway include, among others, regulators of sphingolipid biosynthesis, ceramide synthase complex subunits and aminophospholipid flippases^25–28^. MCC/eisosomes have also been related to Slm-Ypk regulation. Slm proteins have curved F-BAR domains that may account for their targeting to PM furrow-like structures, and their function is necessary for proper eisosome assembly and phosphorylation of the core eisosome components Pil1 and Lsp1 by kinases of the Pkh/TORC2-Ypk pathway^29–31^. Thus, our current understanding of this pathway relates it to PM composition surveillance in response to stress, PM microdomain organization and the regulation of PM dynamics.

In mammalian cells, as in budding yeast, PtdIns4,5P_2_ acts as a molecular PM marker and is involved in dynamic exo/endocytic processes^32^. However, higher cells possess a regulatory pathway that involves conversion of PtdIns4,5P_2_ into PtdIns3,4,5P_3_ by class I phosphatidylinositol 3-kinase (PI3K), an activity that is absent in *S. cerevisiae*. PtdIns3,4,5P_3_ pools locally generated in response to the activation of diverse PI3K-coupled receptors is an important second messenger that regulates cell growth, proliferation, motility, etc^33^. The main transducer of PtdIns3,4,5P_3_ signaling is the AGC kinase Akt, (or protein kinase B, PKB). Akt is recruited directly to the PM via its PtdIns3,4,5P_3_-specific PH domain and there, like in the case of yeast Ypks, it is phosphorylated by both PDK1 in its T-loop and by the TORC2 complex at its TM and HM for full activation^34^. Akt then phosphorylates and regulates multiple downstream targets, including the TORC1 complex, transcription factors and apoptosis regulators, leading to proliferative and anti-apoptotic responses^35,36^. The PI3K-Akt pathway has been thoroughly explored in the last two decades, given its paramount importance in fields related to human health, such as cancer, insulin response and inflammation^33^.

Previously, we engineered the yeast cell to reconstitute the mammalian PI3K-Akt pathway by heterologous expression^37^. We found that tethering the catalytic subunit of PI3K p110α to the PM, via a C-terminal prenylation signal, caused strong yeast growth inhibition due to the conversion of essential PtdIns4,5P_2_ PM pools into futile PtdIns3,4,5P_3_. However, non-prenylated p110α did not affect yeast viability unless any of the Akt isoforms (Akt1, Akt2 or Akt3) was co-expressed. In the latter setting, p110α/Akt toxicity depended on Akt catalytic activity^38^. Thus, Akt should be inflicting its effect in yeast through phosphorylation of endogenous targets. Here we present evidence that PtdIns3,4,5P_3_-activated Akt short-circuits TORC2 signaling by displacing the endogenous PtdIns4,5P_2_-dependent Slm-Ypk kinase, suggesting a conserved role of AGC kinases in PM homeostasis along evolution.

## Results

### PI3K-activated Akt induces actin-supported membranous structures in yeast

**Figure 1:**
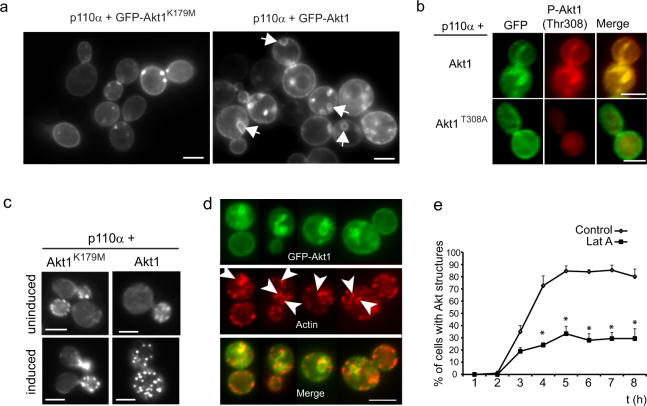
Heterologous GFP-Akt1 activated by PI3K concentrates at actin-supported cytoplasmic compartments in *S. cerevisiae*. (**a**) Fluorescence microscopy on YPH499 yeast cells expressing p110α from plasmid YCpLG-p110α and GFP-Akt1, either a kinase-dead K179M mutant or wild type, from pYES2-based plasmids, as indicated. Arrows point the intracellular compartments decorated by GFP-Akt1. (**b**) Immunofluorescence with anti-GFP and anti-phospho-Akt1 (Thr308) antibodies of yeast cells expressing p110α and either WT GFP-Akt1 or a T308A mutant version, as indicated. (**c**) Actin staining with rhodamine-conjugated phalloidin of cells co-expressing p110α and either WT or the K179M kinase-dead mutant (induced), as above. Cells grown in SC-Raf are shown in the upper panels as a control (uninduced). Representative cells are shown. (**d**) Fluorescence microscopy of cells co-expressing p110α and GFP-Akt1 showing co-localization of actin patches. Arrowheads indicate typical areas of co-localization of red (actin) and green (GFP-Akt1) signals. Four representative cells from the same experiment are shown. In all experiments shown in (**a**–**d**), cells were analyzed after 5 h of galactose induction for expression of the heterologous proteins. All scale bars indicate 5 μm. (**e**) Graph showing the appearance of Akt1-induced intracellular structures expressed in percentage of positive cells in the population over a time-course after induction of p110α and GFP-Akt1 with galactose in the absence (Control) or presence of 80 μM latrunculin A (Lat A). Data are the average of three different experiments (n = 100 cells counted per time point for each experiment). Asterisks (*) indicate statistical significance (p < 0.01 according to Student t-test).

We previously reported that reconstitution of the PI3K-Akt pathway in yeast led to MAPK pathways activation and impaired growth. GFP-Akt1 was recruited to the yeast PM by PtdIns3,4,5P_3_ generated *in situ* by co-expressed PI3K p110α catalytic subunit, a phenomenon dependent on Akt PH domain^37,38^. As shown in Fig. 1a, besides PM localization, cells expressing GFP-Akt1 from the galactose-inducible *GAL1* promoter displayed large Akt1-enriched intracellular compartments. This was not observed in cells expressing kinase-dead GFP-Akt1^K179M^, which just concentrated at small dots. We had previously demonstrated that the yeast PDK1 orthologues, Pkh1 and Pkh2, are responsible for phosphorylation and activation of heterologous Akt1 in its T-loop (Thr308)^37^. Immunofluorescence with specific anti-phospho-Akt1(Thr308) antibodies showed that it was the active form of GFP-Akt1 which accumulated at these large compartments (Fig. 1b, upper panel). Moreover, a non-activatable GFP-Akt1^T308A^ mutant version failed to produce these structures although it was efficiently recruited to the PM by PI3K-generated PtdIns3,4,5P_3_ (Fig. 1b, lower panel). Thus, concomitant PI3K- and Pkh-dependent Akt1 activation is required for the development of intracellular Akt-enriched structures.

Actin staining with fluorochrome-conjugated phalloidine of yeast cells co-expressing mammalian PI3K and Akt1 revealed an abnormal actin distribution: restriction of actin patches to the growing bud was lost in cells expressing wild type –but not kinase-dead-Akt1 (Fig. 1c). Moreover, actin patches co-localized with GFP-Akt1-enriched structures (Fig. 1d). The proportion of cells containing such structures was significantly reduced by treatment with the actin-depolymerizing drug latrunculin A (Fig. 1e). Thus, the generation of Akt1-induced structures relies on actin dynamics.

### Akt1-induced structures are PM invaginations

**Figure 2:**
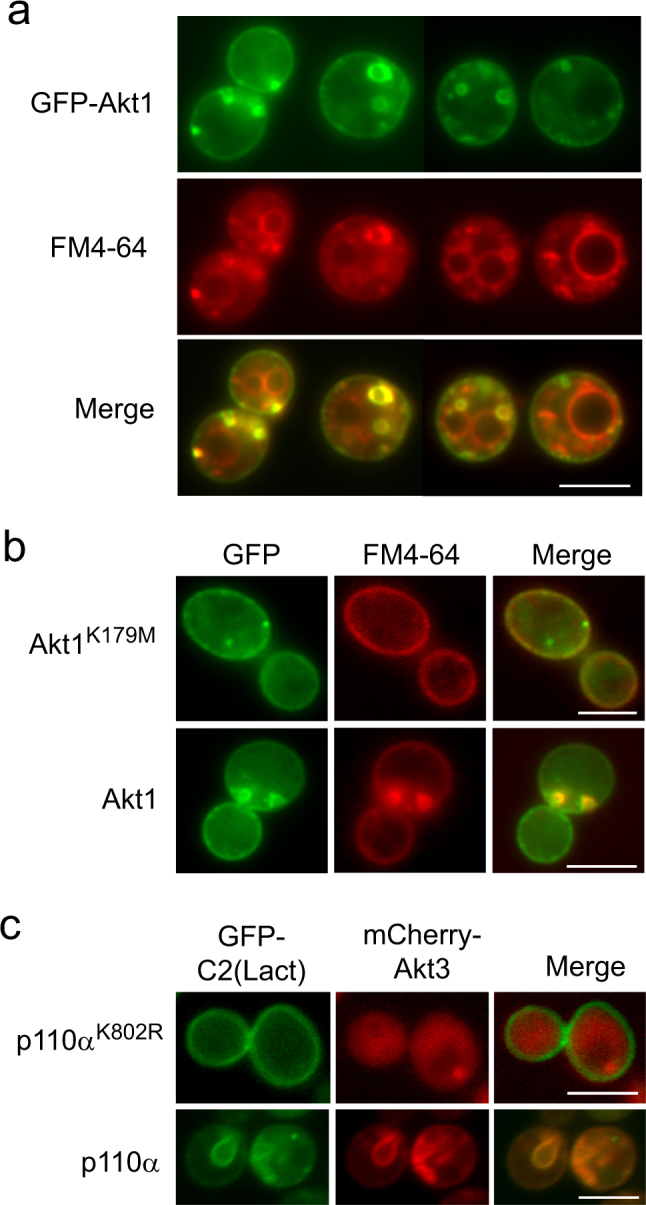
Akt-induced structures are PM invaginations (**a**) Fluorescence microscopy of two fields showing four characteristic FM4-64-treated YPH499 yeast cells cultured in SR-Gal expressing GFP-Akt1 from pYES2-GFP-Akt1 and p110α from YCpLG-p110α. (**b**) Staining of the PM with FM4-64 upon inhibition of endocytosis on either control cells expressing kinase-dead (K179M mutant, upper panel) or wild type GFP-Akt1 (lower panel), both from pYES2-GFP-based plasmids, and co-expressing p110α from YCpLG-p110α. Cells were induced for expression as in (**a**), but incubation with FM4-64 was done at 0 °C in the presence of NaN_3_ and NaF. (**c**) Co-localization of Akt with the PS marker GFP-C2(Lact). Triple transformants bearing pRS410-GFP-LactC2, YCpLG-p110α-CAAX (or a kinase-dead K802R version as a negative control for Akt1 activation; upper panel), and pYES3-mCherry-Akt3 were induced and analyzed as in (**a**). All scale bars indicate 5 μm.

To characterize the nature of Akt1-marked compartments, we used the lipophilic vital dye FM4-64, which binds PM lipids and is internalized by endocytosis, thus labeling compartments along the endocytic pathway until it reaches the vacuolar membrane^39^. As shown in Fig. 2a, in cells co-expressing p110α and GFP-Akt1, GFP fluorescence did not co-localize with FM4-64 at the vacuole after 30 min of incubation. Instead, GFP-Akt1-marked structures matched with intense smaller FM4-64-labeled compartments. Blocking endocytosis by incubating cells on ice in the presence of azide as a metabolic inhibitor leads to persistent PM staining by FM4-64^39^. Interestingly, in these conditions, GFP and FM4-64 co-stained both the PM and active Akt-induced compartments (Fig. 2b), suggesting that such structures corresponded to intracellular PM extensions. In support of this idea, a specific probe for phosphatidylserine (PS) at the PM inner layer, GFP-C2(Lact)^40^, co-localized with mCherry-Akt3 in the presence of p110α, but not when co-expressed with kinase-dead p110α (Fig. 2c). These results indicate that the compartments in which activated Akt accumulated were large PM invaginations.

### Akt-induced PM invaginations promote intracellular cell wall deposition

**Figure 3:**
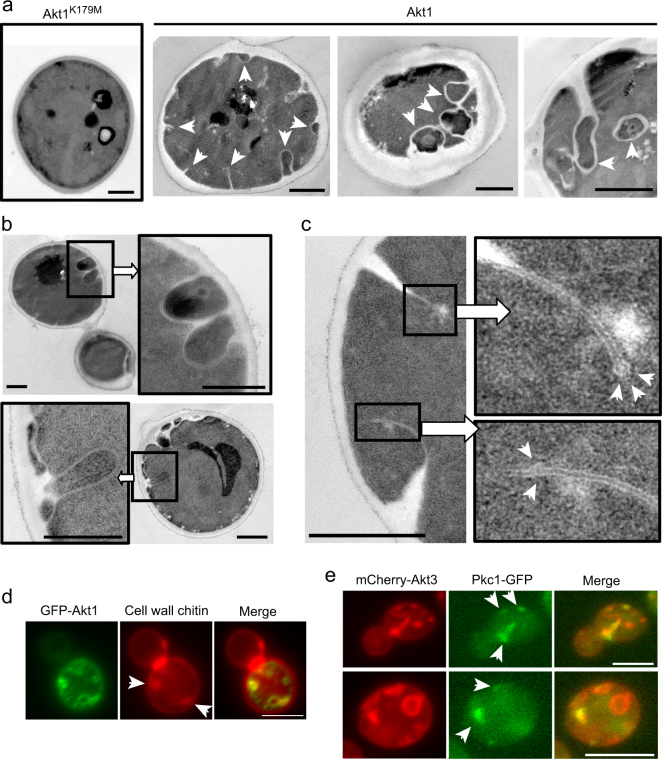
Characterization of Akt-triggered PM invaginations. (**a**–**c**) Transmission electron microscopy (TEM) of representative YPH499 yeast cells expressing p110α from YCpLG-p110α and either kinase-dead GFP-Akt1^K179M^ (a; leftmost panel image) or wild type GFP-Akt1 (the rest) from pYES2-based plasmids, after 5 h of incubation in SR-Gal for induction. In (**a**) three characteristic cells are shown displaying multiple PM invaginations (arrows); the two images on the right display insulated areas within the cytoplasm surrounded by cell wall material. As evidenced by the close-up images in (**b**), such insulated areas seem to develop initially by inwards PM growth, extension and enclosing. The cell in (**c**) shows two invagination events revealing that the section of membrane folds expanding inwards has a rounded tip, reminiscent of pre-scission endocytic vesicles (upper close-up); in the lower close-up, the expanding rounded tip seems to meet a second growing membrane (arrows), perhaps a pre-fusion event that will lead to the enclosing of a cytoplasmic isle such as those shown in (**b**). (**d**) Calcofluor white (CFW) staining to visualize chitin-rich cell wall areas on transformant cells as above. An artificial red color has been used to visualize CFW in this image for better contrast. Patchy, but not bubble-like GFP-Akt1 structures are intensely co-stained by CFW, indicating cell wall deposition (arrows), in consistence with the Akt1-induced phenomena shown by TEM in (**a**–**c**). (**e**) Concentration of GFP-Pkc1 in discrete Akt1-induced structures. Representative cells of MML50 strain (expressing Pkc1-GFP) co-transformed with YCpLG-p110α and pYES3-mCherry-Akt3. Induction of the *GAL1* promoter by incubation in SR-Gal for 5 h was performed prior to observation. Bars represent 1 μm in (**a**–**c**) and 5 μm in (**d**,**e**).

Next, we performed transmission electron microscopy (TEM) on yeast cells co-expressing p110α and Akt1. As shown in Fig. 3, these cells displayed multiple membrane invaginations conforming cytoplasmic islands associated with the cell surface. These structures were frequently paired or concentrated at particular areas of the cell surface (Fig. 3a,b). The observation of invaginating membranes that do not form yet a closed surface-associated compartment (Fig. 3c) suggested that extended folds of membrane expand inwards until they find again the PM and fuse, thus originating cytoplasmic islands. Remarkably, electron-translucent cell wall (CW) polysaccharides were deposited filling some PM invaginations. None of these phenomena were observed in control cells co-expressing p110α and kinase-dead Akt1 (Fig. 3a).

We stained p110α and GFP-Akt1 co-expressing cells with calcofluor white (CFW) to visualize chitin-rich CW regions, to confirm that CW material was accumulated at invaginations. In agreement with TEM observations, some but not all GFP-Akt1 PM extensions were stained with CFW (Fig. 3d). CW growth is supported by actin-driven polarized secretion involving local activation of the Rho1 small GTPase^41^. The localization of the Rho1-activated protein kinase C Pkc1 serves as a spatial marker for sites of Rho1 activation^42,43^. Interestingly, abnormally located GFP-Pkc1 co-localized with some mCherry-Akt3 invaginations (Fig. 3e).

### PtdIns4,5P_2_ is found at Akt-induced membrane invaginations

**Figure 4:**
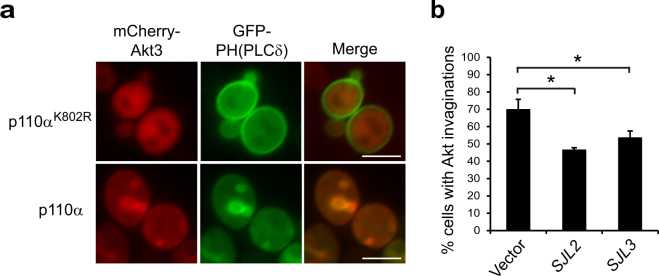
Akt-induced PM invaginations are enriched in PtdIns4,5P_2_. (**a**) Fluorescence microscopy of representative YPH499 cells expressing GFP-PH(PLCδ) from pESC-TRP-GFP-2x-PH(PLCδ) and mCherry-Akt3 from pYES3-mCherry-Akt3, together with either YCpLG-p110α-CAAX (or a kinase-dead K802R version as a control; upper panel). Scale bars indicate 5 μm. (**b**) Overproduction of Sjl phosphatases reduces the appearance of Akt-induced PM invaginations. YPH499 cells were co-transformed with pYES3-GFP-Akt1, YCpLG-p110α and BG1808-SJL2, BG1808-SJL3 or an empty vector as a control. Transformants were incubated for 5 h in SG. Over 100 cells were counted in triplicate per experiment and error bars correspond to the standard deviation. Asterisks (*) indicate statistical significance (p < 0.01 according to Student’s t-test).

In yeast, PtdIns4,5P_2_ plays important roles in endocytosis^44^. PM invaginations similar to those induced by Akt1 have been previously reported in cells lacking synaptojanin-like (Sjl) PtdIns 5-phosphatases, as a consequence of the up-regulation of this phosphoinositide^45,46^. Consistently, we found intense staining of Akt-induced invaginations by the PtdIns4,5P_2_-specific fluorescent probe GFP-PH(PLCδ)^46^, which co-localized with mCherry-Akt3 (Fig. 4a). The presence of PtdIns4,5P_2_ in the invaginations seems paradoxical because Akt localization and activation requires the conversion of PtdIns4,5P_2_ into PtdIns3,4,5P_3_ by co-expressed PI3K. Nevertheless, overexpression of either the Sjl2/Inp52 or Sjl3/Inp53 PtdIns 5-phosphatases partially counteracted the formation of Akt-induced PM invaginations (Fig. 4b), supporting that Akt1 activation by PtdIns3,4,5P_3_ locally increases the levels of its precursor PtdIns4,5P_2_.

### Overexpression of PtdIns4,5P_2_ effector Slm1 mimics the effects of Akt on the PM

**Figure 5:**
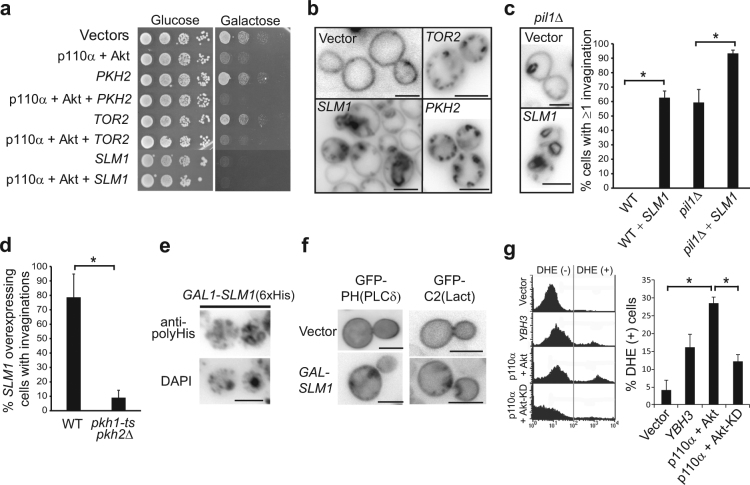
Overexpression of Slm1 leads to PM invaginations similar to those induced by Akt1. (**a**) Representative growth assays of YPH499 cells overexpressing the indicated genes on SD (Glucose; promoter off) and SG (Galactose; promoter on) agar. Transformants bear YCpLG-p110α and pYES3-GFP-Akt1 plasmids, and a third *URA3-*based plasmid either empty or harbouring *PKH1*, *TOR2* or *SLM1* genes. (**b**) Fluorescence microscopy of representative YPH499 transformants as in (**a**), after 5 h of induction in SR-Gal media, following incubation with FM4-64 on ice with NaN_3_ and NaF to inhibit endocytosis. Images were inverted for a better contrast. Bars represent 5 µm. (**c**) *SLM1* overexpression and *pil1Δ* have additive effects. Either wild type BY4741 or isogenic *pil1*Δ*::kanMX4* cells transformed with an empty vector or plasmid BG1805-*SLM1* were cultured for 5 h in SR-Gal. The graph shows the percentage of cells displaying ≥ one invagination after FM4-64 PM staining as in (**b**). (**d**) Pkh function is necessary for the appearance of PM invaginations upon Slm1 overproduction. Either wild type DLY1 or INA106 (*pkh1-ts pkh2*Δ) cells transformed with BG1805-*SLM1* were induced in SR-Gal at 37 °C for 5 h and stained with FM4-64. In (**c**,**d**) >100 cells were counted in triplicate. Error bars correspond to standard deviation. Asterisks (*) indicate statistical significance (p < 0.01; Student’s t-test). (**e**) Immunofluorescence with anti-polyHis antibodies on YPH499 transformants overexpressing Slm1*-*6xHis from vector BG1805-*SLM1* after incubation for 6 h in SR-Gal. Nuclear DAPI staining of the same field is shown. (**f**) Accumulation of PtdIns4,5P_2_ and PS in PM invaginations upon *SLM1* overproduction. Fluorescence microscopy of representative YPH499 cells co-transformed with an empty vector or BG1805-*SLM1* and either pESC-TRP-GFP-2xPH(PLCδ) (left) or pRS410-GFP-LactC2 (right) after 6 h incubation in SR-Gal. (**g**) Graph showing the DHE-positive cell population by flow cytometry (gated as in the representative experiment shown at the left) in YPH499 yeast transformed with YCpLG-p110α and pYES2-GFP-Akt1 (either wild type or kinase-dead K179M mutant, Akt-KD). Negative controls were transformants with pYES2-GFP. Positive controls were *YBH3* transformants. Results are the average from three different clones for each transformant. Error bars correspond to standard deviation. Asterisks (*) indicate statistical significance (p < 0.001; Student’s t-test).

Redundant Slm1 and Slm2 are PtdIns4,5P_2_-binding proteins involved in the TORC2 pathway^23,24^. They recruit Ypk1 and Ypk2 kinases to the PM for their activation by Pkh1/Pkh2 and the TORC2 protein kinase^47^. Our results suggest that PtdIns4,5P_2_-dependent signaling is hyperactivated by heterologous Akt. Thus we investigated whether overproduction of elements of the TORC2 pathway (the Pkh2 kinase; TORC2 components Avo1, Avo2, Avo3 and Tor2; downstream AGC kinases Pkc1, Sch9 and Ypk1; and the Slm1 adaptor) led to effects similar to those caused by Akt. We also overproduced TORC2-signaling targets, like ceramide synthases Lac1 and Lag1^27^, and MCC/eisosome components Lsp1, Pil1 and Sur7^12^, because MCC/eisosomes are also related to PtdIns4,5P_2_ regulation^11,48^. As shown in Table S1 and Fig. 5a, overexpression of Pkc1 and Slm1 was highly toxic for yeast cells. Among the other proteins overexpressed, only Lac1 and Lag1 faintly inhibited yeast growth. When co-expressed with p110α-Akt, only Pkh2 and Slm1 showed a negative genetic interaction, as their overexpression slightly enhanced Akt-induced growth inhibition (Fig. 5a). Remarkably, Slm1 overproduction led to large PM invaginations, reminiscent of those triggered by p110α-Akt (Fig. 5b), in around 60% of cells (Fig. 5c). Although overproduction of Pkh2, Ypk1 and TORC2 components also led to the appearance of PM invaginations (Table S1), they were smaller and less conspicuous than those induced by Slm1- or Akt-overexpressing cells (Fig. 5b).

Eisosome disorganization by absence of Pil1 has also been reported to promote PM invaginations^12^. In our hands, around 60% of *pil1*Δ cells displayed at least one invagination (Fig. 5c), but not those lacking other eisosome components (*lsp1*Δ or *sur7*Δ) (Table S1). Interestingly, this phenotype was significantly enhanced when the *SLM1* gene was overexpressed in a *pil1*Δ background (Fig. 5c). PM invaginations induced by *SLM1* overexpression, like those triggered by Akt, were dependent on Pkh1/2 function, as their induction was significantly diminished in a *pkh1-ts pkh2*Δ strain at the restrictive temperature (Fig. 5d). Also, like Akt, overproduced Slm1 accumulated at these structures, as revealed by immunofluorescence on cells expressing poly-His-tagged Slm1 (Fig. 5e). Furthermore, Slm1-induced structures also contained PS and PtdIns4,5P_2_, as determined by using fluorescent GFP-C2(Lact)-and GFP-PH(PLCδ) probes, respectively (Fig. 5f). As expected, TEM analysis of *SLM1*-overexpressing cells revealed PM extensions into the cytoplasm, occasionally filled with CW material (Fig. S1a). We also found that the number and size of cytoplasmic CW inclusions upon *SLM1* overexpression was enhanced in a *pil1*Δ background (Fig. S1b). Therefore, loss of eisosome function, overproduction of Pkh kinases, TORC2 components or, especially, Slm1 caused PM disturbances reminiscent of local Akt activation, indicating that this mammalian protein could be taking over the yeast TORC2 pathway.

### Akt activation leads to oxidative stress in yeast cells

TORC2 signaling has been reported to influence both PM homeostasis and cytoskeletal regulation via reactive oxygen species (ROS)^49^. We analyzed ROS production by dihydroethidium (DHE) staining and flow cytometry in cells co-expressing p110α with either Akt1 or Akt1^K179M^. Overexpression of the *YBH3*, a gene coding for *S. cerevisiae* BCL-2 Homology domain 3 (BH3)-containing protein^50^ was used as a positive control for ROS generation. As shown in Fig. 5g, Akt1 co-expressed with p110α led to a higher percentage of ROS-producing cells than the kinase-dead Akt1 version, and did so more efficiently than overproduction of Ybh3.

We also performed a transcriptomic analysis to study Akt-induced transcriptional response. Global mRNA expression of cells expressing either p110α with Akt1 or kinase-dead Akt1^K179M^ were compared. Table S2 show the 81 genes that were up-regulated over 1.7-fold and 39 genes down-regulated below 0.6-fold. Genecodis and GO Term Finder analyses on up-regulated genes highlighted three significantly enriched functional categories: “pentose phosphate shunt” (p-value = 9×10^−15^ by chi square test with Bonferroni correction), “oxidation-reduction” (p = 2×10^−4^) and “cell wall organization” (p = 0.05). In down-regulated genes, two functional categories were underscored: “mitotic cell cycle” (p = 2×10^−4^) and again “cell wall organization” (p = 0.01) (Fig. S2a). We then compared our dataset of Akt-induced genes to reported datasets related to oxidative stress (treatment with diamide), cell wall stress (treatment with Congo red) and inhibition of Tor2. Hits from our microarray significantly overlapped with these datasets (56.5%, 43.2% and 22.9%, respectively; Fig. S2b), in agreement with the previously reported activation of the yeast cell wall integrity (CWI) pathway by Akt^38^, as well as the above-described induction of ROS production and alteration of TORC2 signaling. The “pentose phosphate shunt”-related hits were Akt-specific, as it did not overlap with other datasets.

### Akt promotes Slm1 phosphorylation but not TORC2-dependent Ypk1 phosphorylation

**Figure 6:**
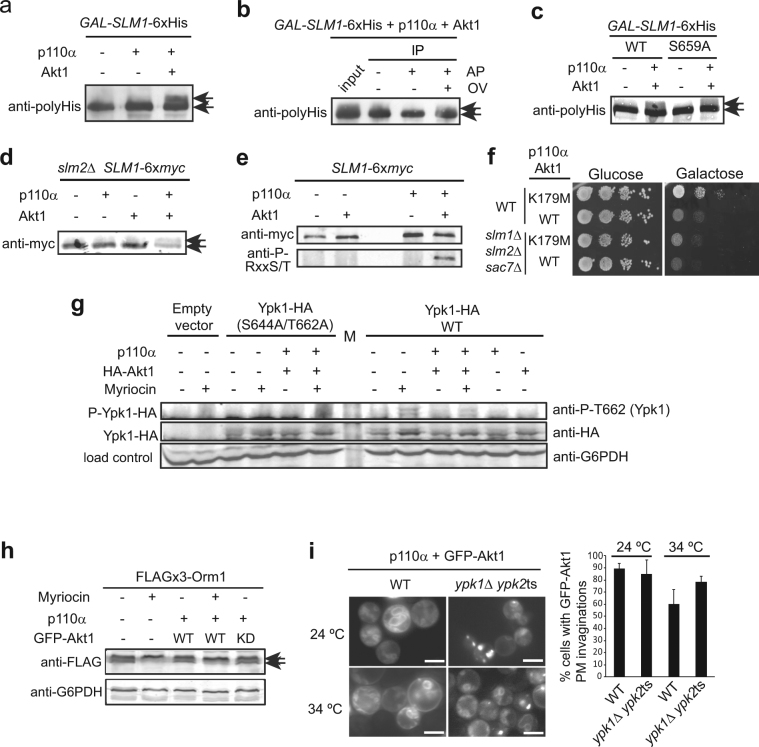
Akt phosphorylates Slm1 but its effects are independent of TORC2-Slm-Ypk signaling. (**a**) Immunoprecipitation of Slm1-6×His expressed from plasmid BG1805-*SLM1* in wild type YPH499 cells co-expressing the empty vectors YCpLG and pYES3 and/or YCpLG-p110α and pYES3-GFP-Akt1. Arrows indicate the two electrophoretic mobility bands of Slm1-6×His observed with anti-polyHis antibody. (**b**) Input and immunoprecipitation (IP) of Slm1-6×His from cell lysates co-expressing BG1805-*SLM1*, YCpLG-p110α and pYES3-GFP-Akt1. Immunoprecipitates were treated with alkaline phosphatase (AP) and additionally with ortovanadate (OV) to inhibit phosphatase activity. (**c**) Immunoprecipitation experiments as in (**a**) but using the non-phosphorylatable Slm1^S659A^ mutant. (**d**) Immunoprecipitation of Slm1-6×Myc, endogenously expressed from strain IRE12 (*SLM1*-6×*myc slm2*Δ), co-transformed with the empty vectors and/or plasmids YCpLG-p110α and pYES3-GFP-Akt1 and immunodetected with anti-Myc antibody. (**e**) As in (**d**), but Slm1-6×Myc was expressed from strain IRE11 and immunoprecipitates were blotted with anti-(R/K)X(R/K)XX(pT/pS) motif (anti-p-Akt-substrate) antibodies. (**f**) Growth assays of wild type (SEY6210) and AAY1663 (*slm1*∆ *slm2*∆ *sac7*∆) cells transformed with YCpLG-p110α and pYES2-GFP-Akt1^K179M^ or pYES2-GFP-Akt1 cultured in SD (Glucose) and SG (Galactose). (**g**) Western-blotting of cells (YPH499) co-expressing the PLB187 (empty plasmid), PLB215 (Ypk1-HA) and PLB534 (Ypk1^S644A, S662A^-HA) and YCpLG/YCpLG-p110α and/or pYES3/pYES3-HA-Akt1. Cells were induced in SR-Gal for 3 h, and, where indicated, treated with 1.5 µM myriocin for 2 additional hours. Extracts were probed with anti-phospho-T662 for Ypk1 phosphorylation, anti-HA for Ypk1 immunodetection, and anti-G6PDH as loading control. The ‘M’ lane denotes the Mw marker. (**h**) Western-blotting of lysates from cells (YDB146) co-transformed with the catalytically-inactive mutant YCpLG-p110α^K802R^ (p110α−) or YCpLG-p110α (p110α+) and pYES2-GFP (GFP-Akt1−), pYES2-GFP-Akt1 (WT) or kinase-dead pYES2-GFP-Akt^K179M^ (KD), immunodetected with anti-FLAG or anti-G6PDH as loading control. As in (**g**), cultures were treated, when indicated, with 1.5 µM myriocin. (**i**) Representative fluorescence images and quantitative analyses of PM invaginations in wild type (YPH499) or YPT40 (*ypk1-1ts ypk2*∆) at permissive (24 °C) or restrictive (34 °C) temperatures, co-transformed with YCpLG-p110α and pYES2-GFP-Akt1, after 24 h incubation in SG medium. Data are the average from 3 different fields (n > 30 cells/field). Error bars correspond to standard deviation. Images in (**a**-**e**,**g**,**h**) correspond to cropped blots for conciseness. Full-length blots are presented in Supplementary Fig. S3.

Slm1 is a phosphoprotein and its phosphorylation relies on both Pkh1/2 and TORC2^24,51,52^. Hence, we decided to study the phosphorylation state of Slm1 in the presence of Akt. As shown in Fig. 6a, overexpressed polyHis-tagged Slm1 migrated as a single band. However, when Slm1 was co-overproduced with p110α-Akt1, but not with p110α alone, an additional band of reduced electrophoretic mobility appeared. This mobility shift corresponded to phosphorylation, as it could be eliminated by phosphatase treatment *in vitro* (Fig. 6b). However, a mutant Slm1^S659A^ version, known to lack Pkh1/2-dependent phosphorylation upon stress^52^, displayed the same pattern as wild type Slm1 (Fig. 6c). Thus, this residue was not involved in the post-translational modification detected upon PI3K-Akt expression. To determine whether this phosphorylation occurred on endogenous Slm1, we integrated a *SLM1-*6×*myc* version by gene replacement in a *slm2*Δ mutant so that all cellular Slm function was dependent on tagged Slm1. The electrophoretic shift of Slm1-6×Myc was still observed (Fig. 6d). Moreover, immunoprecipitation with anti-myc antibodies and immunodetection with anti-phospho-Akt substrate [(R/K)X(R/K)XX(pT/pS)] antibodies showed that Slm1 was recognized by these antibodies only in the presence of Akt1 (Fig. 6e).

The above results led us to hypothesize that the effect of Akt was due to hyperactivation of the Slm-dependent TORC2-Ypk pathway. In that case, the lack of Slm proteins would attenuate Akt-mediated toxicity in yeast. To test this, we used a triple *slm1∆ slm2∆ sac7∆* mutant strain, because the absence of the Rho1 GTPase-activating protein Sac7 counteracts the lethality of a *slm1∆ slm2∆* double mutant^23^. However, although growth of this strain in galactose was deficient, Akt1 still had an inhibitory effect as compared to kinase-dead Akt1 (Fig. 6f), suggesting that Slm function was not determinant for Akt effects in yeast.

Ypk kinases are activated when the TORC2 pathway is stimulated by defects in sphingosine biosynthesis, so treatment with myriocin, a serine palmitoyltransferase inhibitor, is commonly used to activate this pathway^25,53^. To test whether Akt activation led to Ypk1 phosphorylation by TORC2, we analyzed cell lysates expressing Ypk1-HA by immunoblotting with anti-phospho-Ypk1(Thr662)^47^. As a control we used cells expressing the non-phosphorylatable version Ypk1^S644A,T662A^-HA^22^. As shown in Fig. 6g, whereas myriocin led to Ypk1-HA Thr662 phosphorylation, co-expression of p110α and Akt1 in the absence of this compound did not. Thus, in spite of inducing Slm1 phosphorylation, Akt is not promoting Ypk1 phosphorylation.

We also tested whether Akt was able to phosphorylate known Ypk1 substrates, such as Orm1/2 proteins. In response to low sphingolipid levels, these negative regulators of sphingolipid biosynthesis are inactivated by TORC2-activated Ypk1^26^. Treatment with myriocin triggers a electrophoretic mobility shift of 3×FLAG-Orm1, while co-expression of p110α and Akt1 did not (Fig. 6h). This result was consistent with the lack of Ypk1 Thr662 phosphorylation observed in Akt-expressing cells. We also checked whether the production of Akt-induced PM invaginations was dependent on Ypk activity. As shown in Fig. 6i, p110α-Akt1 co-expression still led to the appearance of invaginations upon reduction of Ypk activity in a *ypk1-ts ypk2*Δ mutant at semi-permissive temperature. The fact that Akt-induced phenotypes mimic TORC2 signaling without involving Slm-Ypk activation suggested that Akt is overriding the pathway downstream TORC2 by taking over, at least partially, Ypk function.

### Akt complements loss of Ypk and TORC2 function

**Figure 7:**
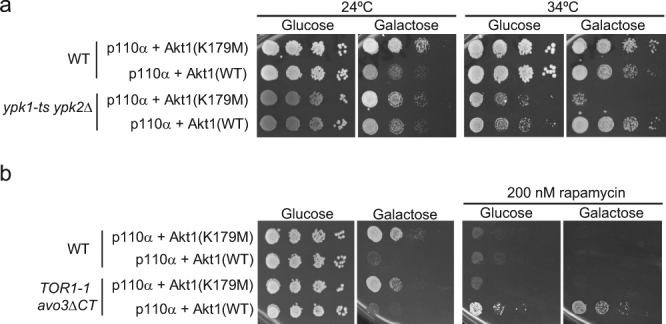
Akt complements the lack of TORC2-Ypk1 function in yeast. (**a**) Growth assays of ten-fold serial dilutions of YPH499 (wild type; WT) and YPT40 (*ypk1-1ts ypk2*∆) cells transformed with YCpLG-p110α and pYES2-GFP-Akt1^K179M^ or pYES2-GFP-Akt1 cultured in SD (Glucose) and SG (Galactose), incubated at 24 °C or 34 °C. (**b**) Growth assays of ten-fold serial dilutions TB50 strain (WT) and mPR8 mutant cells (*TOR1-1 avo3*^∆CT^, otherwise isogenic to TB50) transformed with YCpLG-p110α and pYES2-GFP-Akt1^K179M^ or pYES2-GFP-Akt1 cultured in SD (Glucose) and SG (Galactose) in the presence or absence of 200 nM rapamycin, as indicated.

To test whether Akt is mimicking the essential function of Slm1-Ypk1 downstream TORC2, we performed growth assays with a *ypk1-ts ypk2*Δ mutant expressing p110α with either wild type or kinase-dead Akt1. At the permissive temperature (24 °C), this mutant grew like the wild type and Akt activation negatively affected growth, as expected. However, at the restrictive temperature in galactose-based medium (34 °C), the growth defect of the Ypk-deficient strain was fully relieved by Akt (Fig. 7a). This indicates that Akt can complement a dysfunction of the Slm-Ypk module in the TORC2 pathway. To further prove this point, we tested whether Akt could complement loss of TORC2 function. We used a strain in which TORC2 can be specifically inhibited by rapamycin^54^. Co-expression p110α and Akt, but not the kinase-dead version, indeed rescued the lethality observed upon rapamycin-dependent TORC2 inhibition in galactose-based medium and even partially in glucose-based medium, in which Akt expression should be very low (Fig. 7b).

## Discussion

Eukaryotic cells sense environmental stimuli through PM-located receptors that trigger complex molecular events to reprogram gene expression and protein synthesis. Phosphoinositides are key signal transducing landmarks at cellular membranes. In spite of recent advances, we are only beginning to understand how AGC superfamily protein kinase-mediated pathways respond to lipid second messengers to regulate PM homeostasis upon environmental challenges. In higher cells, one of the most relevant signaling pathways at the PM involves PI3K, which converts PtdIns4,5P_2_ into PtdIns3,4,5P_2_ that locally recruits Akt to phosphorylate multiple substrates. The yeast *S. cerevisiae* is an outstanding model for studies on signaling. Heterologous expression of mammalian PI3K p110α catalytic subunit efficiently produces PtdIns3,4,5P_3_, otherwise absent in yeast, leading to recruitment of Akt to the PM where it is fully activated by endogenous conserved PDK1-like (Pkh1/Pkh2) and presumably TORC2 kinases^37,38,47^. We show here that the most outstanding effect of Akt activity in yeast is the development of large and ubiquitous PM invaginations. A similar phenomenon was reported to occur in cells lacking the PtdIns4,5P_2_ 5-phosphatases Sjl1/Inp51 and Sjl2/Inp52^45,46^. During endocytosis, an actin-driven process, elimination of PtdIns4,5P_2_ by these phosphatases from the forming endocytic vesicle is essential for its scission from the PM^44,55^. The reduction of Akt-induced invaginations by treatment with the actin-depolymerizing drug latrunculin suggests that they derive of growing endocytic-like membranes unable to excise from a PtdIns4,5P_2_-rich PM. It seemed paradoxical that a behavior related to high PtdIns4,5P_2_ levels was triggered by Akt, as its activation is subordinated to local removal of PtdIns4,5P_2_ via its conversion to PtdIns3,4,5P_3_ by co-expressed PI3K. However, we found that Akt-induced invaginated membranes, similar to those in *sjl1Δ sjl2Δ* mutants, were heavily marked by the PtdIns4,5P_2_ PH(PLCδ) reporter, and their number decreased when PtdIns4,5P_2_ levels were reduced by *SJL2/SJL3* overexpression. Thus, local Akt kinase activity must be triggering endogenous pathways that upregulate PtdIns4,5P_2_ synthesis, either by activating the Mss4 PtdIns4P 5-kinase or by inhibiting the PtdIns4,5P_2_ 5-phosphatases.

Some PM invaginations induced by Akt were accompanied by abnormal intracellular CW deposition. Both recruitment of Pkc1 to invaginated membranes and transcriptomic data showing a significant differential expression of genes related to CW biogenesis are consistent with local hyperactivation of the Rho1-Pkc1 pathway at these sites to facilitate actin-based polarized secretion and CW deposition. Actually, we had previously reported Akt-dependent activation of the Pkc1-dependent CWI MAPK pathway^38^. Although our static EM analyses cannot establish the order of events at the PM following Akt activation, our data are consistent with the idea that large PM invaginations form prior to CW material deposition on the extracellular side, ultimately leading to their expansion. PM and CW growth into the cell is reminiscent of the sequence of events required for septation, when the PM is invaginated at the bud neck by mechanisms divergent to those governing endocytosis, likely maintaining high levels of PtdIns4,5P_2_ in order to prevent membrane scission^56^.

Remarkably, analogous PM invaginations were observed by growing yeast cells in the presence of 1-*O*-hexadecyl-2-acetyl-sn-glycerophosphocholine, also known as C16:0 Platelet Activating Factor (PAF), a neurotoxic lipid that accumulates in neurons in Alzheimer disease^57^. In contrast to those induced by Akt, such structures were formed independently of the actin cytoskeleton and were not reported to be associated with internal cell wall deposition^57^. However, in spite of these differences, PAF-induced structures were also enriched in PtdIns4,5P_2_ suggesting that both phenomena are caused by remodeling of PM PtdIns4,5P_2_ distribution. Kennedy *et al*. concluded that the accumulation of sphingolipids in PAF-treated cells contributes to the re-localization of PtdIns4P 5-kinase Mss4, leading to an increase in PtdIns4,5P_2_ levels^57^. Thus, Akt activation in yeast could be related to modulation of sphingolipid metabolism as well. Consistently, we observed that treatment with myriocin, an inhibitor of the sphingolipid synthesis pathway, did cause a 30-fold decrease in the formation of Akt-induced PM invaginations (data not shown). How alteration of complex sphingolipids might lead to a concomitant increase in PtdIns4,5P_2_ is unclear, but mounting evidence suggests links between sphingolipid regulation and the TORC2-binding proteins Slm1 and Slm2, which are PtdIns4,5P_2_ effectors^47,51,52^. Consistently, here we show that overexpression of TORC2 complex components, especially Slm1, led to PtdIns4,5P_2_-enriched invaginations reminiscent of those induced by PAF, Sjl phosphatases elimination or activated Akt.

Eisosome complexes are integrated by the BAR domain-containing proteins Pil1 and Lsp1 that assemble at PM MCC microdomains^13^. These compartments are regulated by Pkh kinases^31,58^, contain Slm proteins^30,53^, and play a role in PtdIns4,5P_2_-signaling towards actin-mediated endocytosis^24,52^. Eisosomes participate in the regulation of sphingolipid metabolism^59^ and endosomal scission^60^, as well as in the maintenance of PtdIns4,5P_2_ homeostasis by recruitment of the Sjl1 phosphatase^48^. Accordingly, defects in eisosome organization achieved by elimination of Pil1 are associated with an increase in PtdIns4,5P_2_^48^. Therefore, PM invaginations observed here and reported by other authors in *pil1*∆ mutant cells^12,61^ should also be related to high levels of this phosphoinositide. Consistently, we found that the *pil1*∆ phenotype and the one caused by Slm1 overexpression were additive. Slm proteins have been proposed to shift from MCC/eisosomes to MCT in response to PM stress, thus activating TORC2-Ypk1 signaling to readapt membrane composition to face such situation^26,28,47,53^. Moreover, Slm1 overexpression has been shown to trigger TORC2-Ypk1 activation even in the absence of stress^53^. In view of these data, it is plausible that Slm1 overexpression phenotypically resembles Akt activation by enhancing its presence at the MCT and promoting a local increase in PtdIns4,5P_2_, as happens when MCC/eisosomes are impaired. The fact that Slm1 becomes hyperphosphorylated upon Akt activation suggests their co-existence in particular PM domains. The recognition of Slm1 by antibodies specific to Akt-phosphorylated substrates supports the idea that it is a direct target of heterologous Akt. However, Slm1 sequence lacks a typical RxRxxS/T motif and these antibodies may also recognize phosphorylated minimum RxxS/T motifs that do not necessarily fit the Akt consensus, so we cannot discard that phosphorylation of Slm1 may be indirect through another kinase as a consequence of Akt interference with the TORC2-Ypk pathway.

**Figure 8:**
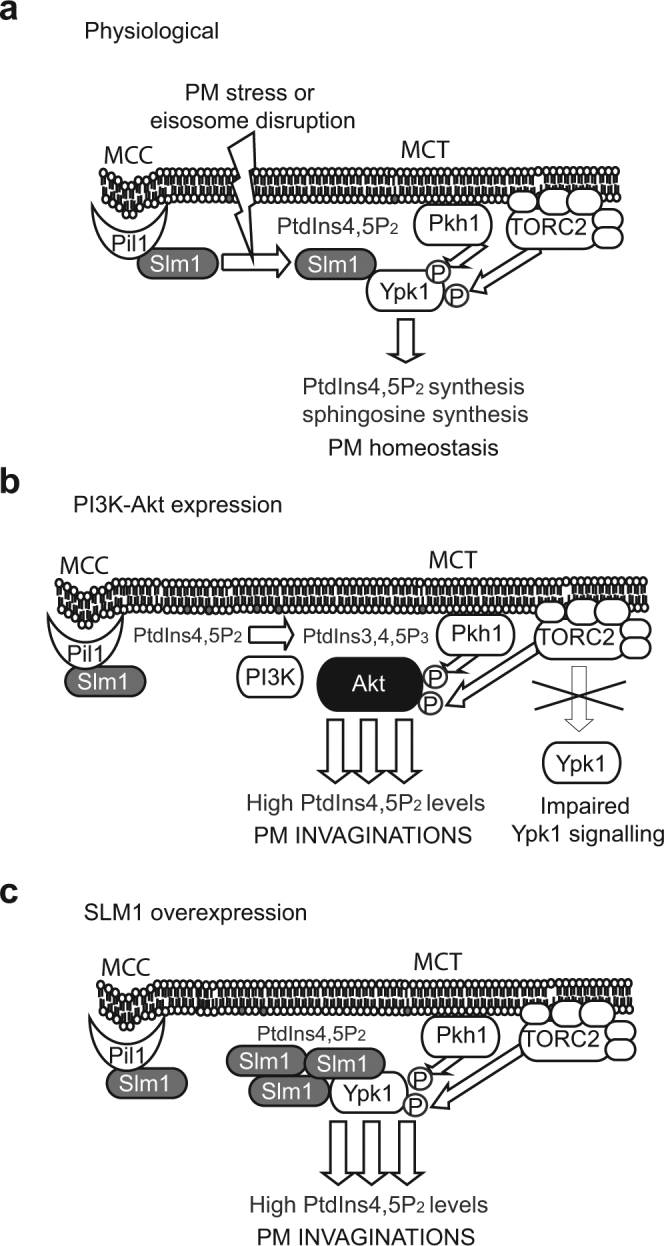
A model for the interference of Akt with PtdIns4,5P_2_ and TORC2 signaling. In physiological conditions (**a**), regulatory complexes at the MCC/eisosomes and MCT/TORC2 microdomains regulate PM PtdIns4,5P_2_ and sphingolipid levels, respectively, in a coordinated way. PM Stress or lipid misbalance cause Slm1 to shift between compartments and bring Ypk1 in proximity to its activating kinases, turning on the TORC2 pathway. When mammalian p110α and Akt1 are co-expressed in yeast (**b**), PM pools of PtdIns4,5P_2_ are converted into PtdIns3,4,5P_3_ by PI3K activity, bringing Akt in proximity with PDK-like Pkh kinases and TORC2. Thus Akt takes over the role of Ypk short-circuiting TORC2 signaling and leading to enhanced PtdIns4,5P_2_ and phosphoinositide-dependent signaling for membrane growth inwards and actin-supported cell wall deposition. Artificial overproduction of Slm1 (**c**) leads to similar effects, probably by enhancing its presence at the MCT and biasing Ypk signaling towards a PtdIns4,5P_2_-dependent response uncoupled from physiological TORC2 modulation.

The role of Slm1 in the TORC2 pathway is the recruitment of Ypk kinases in proximity of its activators: the TORC2 complex and yeast PDK1 orthologs Pkh1/2^47,53^ (Fig. 8a). Although yeast Ypk kinases have been proposed to be presumptive ortologues to mammalian SGK and/or Akt^45,62^, it has been demonstrated that Ypk kinases can be replaced by SGK but not Akt^20^. However, we show here that, when activated by PI3K, Akt is indeed capable of covering the essential functions of Ypk1/2 and TORC2 kinases in yeast. In these conditions, the presence of a PH domain in Akt, which is absent in Ypks, avoids a requirement for Slm1 to interact with the yeast PM. By this means, PI3K-activated Akt takes over the function of the Slm-Ypk complex on selective targets related to PtdIns4,5P_2_-dependent signaling (Fig. 8b), uncoupling PtdIns4,5P_2_ from sphingolipid-dependent signaling, an effect that can be mimicked by Slm1 overexpression (Fig. 8c). This situation seems to interfere with physiological TORC2-Ypk function on sensing PM stress, as Akt did neither stimulate TORC-dependent phosphorylation of Ypk1 nor activated known TORC2-Ypk1 substrates, such as Orm1^26^. Moreover, the yeast transcriptomic profile of Akt-expressing cells partially overlapped that of a *tor2*-Ts mutant shifted to restrictive temperature. In addition, it has been reported that TORC2-Ypk1 must be activated in order to suppress ROS accumulation^49^, which could explain the significant increase in cellular ROS levels observed upon Akt activation in yeast. Also, loss of TORC function has been related to inefficient endocytic scission, mislocalization of the phosphatase Sjl2 and a consequent increase in PM PtdIns4,5P_2_ levels^63^, phenotypes reminiscent of Akt activation. Finally, the aforementioned PtdIns4,5P_2_-enriched invaginations induced by PAF have also been associated with inhibition of TORC2^57^. Therefore, although Akt is able to complement TORC2-Ypk loss of function, it seems to negatively interfere with the physiological activation of this pathway at the PM.

In sum, we report here that Akt activation in yeast short-circuits endogenous signaling pathways by taking over the function of Ypk kinases in the TORC2 pathway, underscoring the importance of phosphoinositides and downstream kinases in the control of PM homeostasis in all eukaryotes. Therefore, heterologous expression of Akt provides a means to studying its activity in a simple model while it offers a tool for studying phosphoinositide- and TORC2-dependent signaling in yeast.

## Methods

### Strains, media and growth conditions

The *S. cerevisiae* strains used in the present study were YPH499 (*MATa ade2-101 trp1-63 leu2-1 ura3-52 his3-200 lys2-801*); BY4741 (*MATa his3*Δ*1; leu2*Δ*; met15*Δ*; ura3*Δ) (EUROSCARF); MML50 (*MATα leu2-3,112 ura3-52 trp1 his4 can1*^*R*^*; PKC1-GFP::kanMX4*)^64^; Y06988 (*pil1*∆) derives from BY4741 background and carry the corresponding gene completely deleted and replaced by the Geneticin resistance-codifying *KanMX4* module (EUROSCARF); DLY1 (*MATa ade1 his2 leu2-3,112 trp1-1 ura3*Δ); INA106 (DLY1 *pkh1*^D398G^ *pkh2::LEU2*)^65^; and YPT40 (YPH499 *ypk1-1ts*::*HIS3 ypk2-∆1*::*TRP1*)^20^. Strains SEY6210 and AAY1663 are described by Robinson *et al*.^66^ and Audhya *et al*.^23^, respectively. Strains TB50 (*MATa leu2 ura3 rme1 his3Δ*) and isogenic mPR8 [*MATα tor1-1 avo3Δ1274-1430::hphMX6*] was described in Gaubitz *et al*.^54^. Strain YDB146 (BY4741, *3XFLAG-ORM1*) is described by Breslow *et al*.^67^.

Strain IRE12 (isogenic to RL136-1a strain^68^, except *SLM1-6MYC URA3*), was constructed by allelic substitution. A fragment of *SLM1* with was amplified by PCR using oligonucleotides Slm1-UP (5′-GGATGCGCGCTATTCAG-3′) and Slm1-LO (5′-CGGGATCCATGATGGTGATGATGATG-3′), by using plasmid BG1805-*SLM1* as template (Yeast ORF collection, GE Healthcare), and further subcloning into the *Bam*HI sites of the pRS306-6myc vector (gift of H. Martin), thus generating a C-terminal fusion to six Myc epitope copies. Then, this plasmid was digested with *Sph*I and transformed into RL136-1a strain. The strain IRE11 (isogenic to YPH499, *SLM1-6myc URA3*) was constructed by the same strategy.

The general non-selective medium for yeast cell growth was YPD [1% yeast extract, 2%peptone and 2% glucose] broth or agar. For transformation and plasmid maintenance, we used Synthetic Dextrose media (SD; 2% glucose, 0.17% yeast nitrogen base without amino acids, 0.5% ammonium sulphate and 0.12% of synthetic amino acid drop-out mixture, lacking appropriate amino acids and nucleic acid bases to maintain selection for plasmids). For induction of the *GAL1* promoter, synthetic galactose (SG) and synthetic raffinose (SR) media were used, in which glucose was replaced with 2% galactose or 1.5% raffinose, respectively. *GAL1* induction in liquid media was performed by growing cells in SR to mid-exponential phase and then refreshing the cultures to an OD_600_ of 0.3 directly with SG or with SR supplemented with galactose 2% (SR-Gal) for 5–8 h. Growth drop assays on plates were performed as described^37^.

### Plasmids

Transformation of *E. coli* and yeast and other basic molecular biology methods were carried out using standard procedures. Plasmids YCpLG-PI3K, YCpLG-p110-CAAX, pYES2-GFP-c-Akt1, pYES2-GFP-c-Akt1^K179M^ and pYES3-GFP-Akt1 have been already described^37,69^. Plasmids overexpressing Sjl2, Sjl3, Slm1, Pkh1, Pkh2, Ypk1, Sch9, Tor2, Avo1, Avo2, Avo3, Lac1, Lag1, Pil1, Lsp1, Sur7 and Ybh3 were obtained from the Yeast ORF collection (GE Healthcare). They are all based in the backbone *GAL1* vector BG1805. Plasmid BG1805-*SLM1*^S659D^ was constructed by site-directed mutagenesis followed by *Dpn*I-digestion using primers Slmmut-1 (5′-CAAATCCGAATACATCCATGTCTGCATTACCTGATACTAATGATTCTG-3′) and Slmmut-2 (5′-CAGAATCATTAGTATCAGGTAATGCAGACATGGATGTATTCGTATTTG-3′).

pYES3-mCherry-c-Akt3 was constructed as follows: first, we obtained the pYES3-mCherry plasmid by PCR amplification of the sequence of mCherry with primers containing *Hind*III/*Bam*HI restriction sites respectively (5′-GAAGCTTCCCGGGGTGAGCA-3′ and 5′-GGGGATCCTTACTTGTACAGCTCGTC-3′) using as template pCRII-TOPO^TM^-mCherry (a gift from Dr. Peñalva, CIB-CSIC, Madrid). Then, *AKT3* was subcloned from pYES2-GFP-AKT3^38^ into *Eco*RI-*Xba*I restriction sites of the pYES3-Cherry plasmid. pYES3-HA-Akt1 plasmid was obtained as follows: the HA-Akt insert from pMSCV-HA-Akt vector (a gift from Dr. I. Vivanco, UCLA, United States) was digested with *Bgl*II-*Eco*RI and inserted into *Bam*HI/*Eco*RI restriction sites of pYES3 plasmid.

pESC-TRP-GFP-2xPH(PLCδ) was constructed by PCR amplification of a GFP N-terminal fusion containing two tandem copies of the PH domain of PLCδ, using plasmid pRS426-GFP-2 × PH(PLCδ) as a template^46^ and subsequent subcloning into *Spe*I/*Bgl*II sites of pESC-TRP (Agilent®). Plasmid pRS410-GFP-LactC2 was a generous gift from Dr. S. Grinstein (Hospital for Sick Children, Toronto, Canada).

Plasmids PLB187 (empty plasmid), PLB215 (Ypk1-HA) and PLB534 (Ypk1^S644A,S662A^-HA)^47^, were kindly provided by Dr. T. Powers (UC Davis, CA, USA).

### Flow cytometry assays

For the analysis of ROS, yeast transformants were grown in SR medium lacking uracil at 30 °C overnight. Then, the cultures were induced with galactose for 16 h and dihydroethidium (2.5 μg/mL) was added for 5 min. Three thousand cells per second were analysed on a FACScan flow cytometer (Becton Dickinson) on the FL2 log scale. WinMDI 2.7 software was used to analyze the graphics obtained.

### Fluorescence microscopy and immunofluorescence

For *in vivo* fluorescence microscopy (GFP and mCherry observation), cells from exponentially growing SR cultures were induced with 2% galactose for 4 h harvested by centrifugation 10,000 rpm 1 min, washed once with phosphate saline buffer and viewed directly. Cells were examined with an Eclipse TE2000U microscope (Nikon) using the appropriate sets of filters. Digital images were acquired with Orca C4742-95-12ER charge-coupled device camera (Hamamatsu) and were processed with the HCImage software (Hamamatsu, Japan). For statistics on cell populations >100 cells were counted for each experiment. Observation of actin in yeast cells with rhodamine-conjugated phalloidin (Sigma, St. Louis, MO, USA) was performed as previously described^43^. Latrunculin A (Sigma, St. Louis, MO, USA) was added at 80 µM at the time of galactose induction and samples were collected each hour and analysed. Labelling with FM4-64 (*N*-[3-triethylammoniumpropyl]-4-[*p*-diethylaminophenylhexatrienyl] pyridinium dibromide; Molecular Probes, Invitrogene) was done essentially as described^39^. Briefly, cells from exponentially growing cultures and induced with galactose were harvested by centrifugation and labelled with 2.4 µM FM4-64, washed in PBS and analyzed. To visualize cells upon endocytosis inhibition conditions, cells were treated with endocytosis inhibitors NaN_3_ and NaF (10 mM each), stained with FM4-64 on ice, and observed^46^. Calcofluor white (CFW) staining was performed by collecting yeast cells and adding CFW (Sigma, St. Louis, MO, USA) to a final concentration of 5 µg/mL. After 10 min of incubation, cells were washed with PBS three times prior to visualization.

Yeast immunofluorescence was performed by standard procedures. Primary antibodies used were anti-Phospho-Akt (Thr308) (Cell Signaling) at a dilution of 1:200 and anti-GFP JL-8 (Clontech) at 1:200. Secondary antibodies were anti-rabbit IgG Alexa Fluor® 488 and anti-mouse IgG Alexa Fluor® 568, respectively, both diluted to 1:500.

### Electron microscopy

After galactose induction (6 h), yeast cells were fixed by adding to the culture an equal volume of 6% paraformaldehyde and 4% glutaraldehyde in 0.2 M potassium phosphate buffer (pH 6.5) and three times in water, and then treated with 1% KMnO_4_ for 2 h on ice, followed by three times of rinses with water. The samples were subsequently dehydrated and then embedded in Spurr’s low viscosity media (EM Science) as described by the manufacturer. Ultrathin sections were cut and examined under a Zeiss EM902 electron microscope.

### Preparation of cell lysates, immunoprecipitation and Western blotting

Overnight cultures of cells carrying *GAL1*-driven expression plasmids growing in SR media were refreshed to an OD_600_ of 0.3 in SR-Galactose 2% in order to achieve *GAL1*-promoter induction. After 5–6 hours of incubation at 30 °C cells, yeast extracts were obtained as previously described^37^. For Ypk1-phosphorylation analysis, the same general procedure was followed but control cells were treated, after 3 h of *GAL1*-induction, with 1.5 µM myriocin (Cayman Chemical, Ann Arbor, MI, USA), for two additional hours as described by Niles *et al*.^47^. For the Orm1 electrophoretic shift assay, control cells were treated, after 4 h of *GAL1*-induction, with 0.4 µM myriocin for 90 additional minutes, as described in Roelants *et al*.^26^. In this particular case, yeast extracts were obtained following TCA precipitation. To this end, TCA was added to the cultures at a final concentration of 2% and they were kept on ice for 20 min. Afterwards they were centrifuged, washed with 10 mM sodium azide, resuspended in 500 µL of pre-chilled TCA buffer (10 mM Tris pH 8.0, 10% TCA, 25 mM NH_4_OAc, 1 mM Na_2_EDTA) and glass beads added. Cells were broken in a FastPrep®-2 4 at 5.5 rpm, during 30 sec for 3 times, chilling on ice in between. Samples were centrifuged and precipitated proteins were resuspended in 75 µL of resuspension buffer (100 mM Tris, 3% SDS, pH 11.0), and boiled at 95 °C for 5 minutes. Samples were centrifuged again to remove cellular debris and clarified lysates were collected to new tubes.

Inmunoprecipitation and alkaline phosphatase treatment were performed as described^70^. For Slm1*-*6xHis immunoprecipitation, anti-polyHistidine (Clone HIS1, Sigma) antibody was used. For dephosphorylation assays, calf intestine alkaline phosphatase (20 Units) and sodium orthovanadate (10 mM) were added.

For endogenous Slm1-6xMyc immunoprecipitation experiments, 300 µL of yeast extracts were incubated with beads (Dynabeads® Protein G, Thermofisher Scientific), which were previously treated overnight with the antibody anti-cMyc 9E10 (1:100, Santa Cruz). After washing three times, beads were resuspended in SDS-PAGE loading buffer and subjected to immunoblot.

Western-blotting analyses were performed following the general procedure described previously^37^. Immunodetection was carried out with anti-phospho-Ypk1 (T662) (1:20000, kindly provided by Dr. T. Powers, UC Davis, CA, USA), anti-HA 12CA5 (1:1000, Roche), anti-FLAG clone M2 (1:1000, Sigma), anti-cMyc 9E10 (1:1000, Santa Cruz), anti Phospho-Akt Substrate [(R/K)X(R/K)XX(pT/pS)] (1:1000, cs 9611 s, Cell Signaling) and anti-polyHistidine (1:1000, Clone HIS1, Sigma).

Secondary antibodies used for Western-blotting analyses were either horseradish peroxidase (HRP)-conjugated anti-mouse secondary antibodies (for blots in Fig. 6a,b), or anti-mouse IgG-Alexa FluorR 680, anti-rabbit IgG-IRDyeR 800 CW and anti-rabbit IgG-IRDyeR 680; all from LI-COR (Lincoln, NE, USA) at 1:5000 dilution (for the rest of the immunoblots). A chemiluminiscence detection system (ECL™; Amersham Biosciences, UK) or an Oddissey infrared imaging system (LI-COR; Lincoln, NE, USA) system were used for developing the Western blots, respectively.

### Accession code

The accession code for the microarray data deposited in Gene Expression Omnibus Database is GSE107482.

## Electronic supplementary material

Supplementary Material
