# Supplementary material for: Heterologous mammalian Akt disrupts plasma membrane homeostasis by taking over TORC2 signaling in Saccharomyces cerevisiae
Source: Sci Rep. 2018 May 16;8:7732. doi: 10.1038/s41598-018-25717-w (PMC5955888; doi:10.1038/s41598-018-25717-w)
Supplement: Supplementary file 1 — Supplementary Material [file 41598_2018_25717_MOESM1_ESM.pdf]

SUPPLEMENTARY MATERIAL

Heterologous mammalian Akt disrupts plasma membrane homeostasis by taking over TORC2 signaling in *Saccharomyces cerevisiae*

Isabel Rodríguez-Escudero, Teresa Fernández-Acero, Víctor J. Cid and María Molina.

Supplementary Tables

Table S1. Characterization of the overexpression of PtdIns4,5P_2 pathway components as compared to PI3K-Akt.

Overexpressed protein	Plasma membrane invaginations*	Toxicity for yeast cells
p110α + Akt1	++++	++
Slm1	++++	+++
Pkh2	++	-
Ypk1	+	-
Sch9	-	-
Pkc1**	-	+++
Avo1	+	-
Avo2	+	-
Avo3	+	-
Tor2	++	-
Lac1	-	+
Lag1	-	+
Pil1	-	-
Lsp1	-	-
Sur7	-	-

*As detected microscopically by staining PM with FM4-64 at 0 ºC in the presence of azide.

**A constitutively active mutant was expressed[1].

Table S2. Genes up- and down-regulated in yeast cells expressing Akt1 as compared to kinase-dead Akt1^{K179M}, both in the presence of mammalian p110α.

**Upregulated genes**				
**GO (Function)**	**ORF**	**Gene**	**Ratio Akt/Akt-KD**	**Description**
**Oxidation-reduction process**	*YDR453C*	*TSA2*	4.73	Stress inducible cytoplasmic thioredoxin peroxidase; cooperates with Tsa1p in the removal of reactive oxygen, nitrogen and sulfur species using thioredoxin as hydrogen donor.
	YAL061W	*BDH2*	4.02	Putative medium-chain alcohol dehydrogenase with similarity to BDH1.
	YCR021C	*HSP30*	3.90	Negative regulator of the H(+)-ATPase Pma1p; stress-responsive protein.
	YKL026C	*GPX1*	3.41	Phospholipid hydroperoxide glutathione peroxidase; induced by glucose starvation that protects cells from phospholipid hydroperoxides and nonphospholipid peroxides during oxidative stress.
	YMR169C	*ALD3*	2.68	Cytoplasmic aldehyde dehydrogenase; involved in beta-alanine synthesis; uses NAD+ as the preferred coenzyme; very similar to Ald2p; expression is induced by stress.
	YKL107W	*YKL107W*	2.49	Putative short-chain dehydrogenase/reductase.
	YKR049C	*FMP46*	2.10	Putative redox protein containing a thioredoxin fold.
	YPL017C	*IRC15*	2.1	Microtubule associated protein; regulates microtubule dynamics; required for accurate meiotic chromosome segregation.
	YJR078W	*BNA2*	1.88	Tryptophan 2.3-dioxygenase or indoleamine 2.3-dioxygenase; required for de novo biosynthesis of NAD from tryptophan via kynurenine.
	YJR096W	*YJR096W*	1.85	Xylose and arabinose reductase; member of the aldo-keto reductase (AKR) family.
	YDL085W	*NDE2*	1.76	Mitochondrial external NADH dehydrogenase; catalyzes the oxidation of cytosolic NADH; Nde1p and Nde2p are involved in providing the cytosolic NADH to the mitochondrial respiratory chain
	YHL021C	*AIM17*	1.71	Putative protein of unknown function; the authentic, non-tagged protein is detected in highly purified mitochondria in high-throughput studies.
**Cell wall organiza-tion**	*YBR076W*	*ECM8*	2.35	Non-essential protein of unknown function.
	YLR120C	*YPS1*	2.27	Member of the yapsin family of proteases, attached to the plasma membrane via a glycosylphosphatidylinositol (GPI) anchor. Involved with other yapsins in the cell wall integrity response.
	YLR194C	*NCW2*	2.23	Structural constituent of the cell wall; attached to the plasma membrane by a GPI-anchor; expression is upregulated in response to cell wall stress.
	YBR005W	*RCR1*	2.13	Protein of the ER membrane involved in cell wall chitin deposition; may function in the endosomal-vacuolar trafficking pathway, helping determine whether plasma membrane proteins are degraded or routed to the plasma membrane.
	YGR032W	*GSC2*	2.03	Catalytic subunit of 1.3-beta-glucan synthase; involved in formation of the inner layer of the spore wall; activity positively regulated by Rho1p and negatively by Smk1p.
	YKR061W	*KTR2*	2.01	Mannosyltransferase involved in N-linked protein glycosylation; member of the KRE2/MNT1 mannosyltransferase family.

	YKL163W	*PIR3*	1.72	O-glycosylated covalently-bound cell wall protein; required for cell wall stability; expression is cell cycle regulated, peaking in M/G1 and also subject to regulation by the cell integrity pathway.
**Meiotic cell cycle**	*YDL222C*	*FMP45*	3.15	Integral membrane protein localized to mitochondria; required for sporulation and maintaining sphingolipid content; similar to SUR7.
	YML128C	*MSC1*	2.13	Protein of unknown function; mutant is defective in directing meiotic recombination events to homologous chromatids.
	YLR054C	*OSW2*	2.08	Protein of unknown function reputedly involved in spore wall assembly.
	YGR059W	*SPR3*	2.02	Sporulation-specific homolog of the CDC3/10/11/12 family of genes; septin protein involved in sporulation.
	YNL194C	*YNL194C*	1.96	Integral membrane protein; required for sporulation and plasma membrane sphingolipid content; similar to SUR7.
**Pentose-phosphate shunt**	*YGR043C*	*NQM1*	3.72	Transaldolase of unknown function.
	YGR256W	*GND2*	3.66	6-phosphogluconate dehydrogenase (decarboxylating); catalyzes an NADPH regenerating reaction in the pentose phosphate pathway.
	YBR117C	*TKL2*	3.04	Transketolase; catalyzes conversion of xylulose-5-phosphate and ribose-5-phosphate to sedoheptulose-7-phosphate and glyceraldehyde-3-phosphate in the pentose phosphate pathway.
	YGR248W	*SOL4*	2.01	6-phosphogluconolactonase; protein abundance increases in response to DNA replication stress.
**Metabolic process**	*YER037W*	*PHM8*	2.27	Lysophosphatidic acid (LPA) phosphatase. nucleotidase; principle and physiological nucleotidase working on GMP. UMP and CMP; involved in LPA hydrolysis in response to phosphate starvation and ribose salvage pathway.
	YDR380W	*ARO10*	2.10	Phenylpyruvate decarboxylase; catalyzes decarboxylation of phenylpyruvate to phenylacetaldehyde, which is the first specific step in the Ehrlich pathway.
	YBR004C	*GPI18*	1.82	Functional ortholog of human PIG-V; PIG-V is a mannosyltransferase that transfers the second mannose in glycosylphosphatidylinositol biosynthesis.
	YMR271C	*URA10*	1.81	Minor orotate phosphoribosyltransferase (OPRTase) isozyme; catalyzes the fifth enzymatic step in the de novo biosynthesis of pyrimidines.
	YKL151C	*NNR2*	1.75	NADHX dehydratase; converts (S)-NADHX to NADH in an ATP-dependent manner.
	YOR237W	*HES1*	1.74	Protein implicated in the regulation of ergosterol biosynthesis; one of a seven member gene family with a common essential function and non-essential unique functions.
	YER054C	*GIP2*	1.71	Putative regulatory subunit of protein phosphatase Glc7p; involved in glycogen metabolism.
	YMR250W	*GAD1*	1.70	Glutamate decarboxylase; converts glutamate into gamma-aminobutyric acid (GABA) during glutamate catabolism.
**Signaling**	*YOR134W*	*BAG7*	3.18	Rho GTPase activating protein (RhoGAP); stimulates the intrinsic GTPase activity of Rho1p, which plays a bud growth by regulating actin cytoskeleton organization and cell wall biosynthesis, resulting in the downregulation of Rho1p.
	YNL093W	*YPT53*	2.68	Stress-induced Rab family GTPase; required for vacuolar protein sorting and endocytosis; involved in ionic stress tolerance.
	YGL248W	*PDE1*	2.03	Low-affinity cyclic AMP phosphodiesterase; controls glucose and intracellular acidification-induced cAMP signalling, target of the cAMP-protein kinase A (PKA) pathway.

	YLR178C	*TFS1*	1.96	Inhibitor of carboxypeptidase Y (Prc1p). and Ras GAP (Ira2p); phosphatidylethanolamine-binding protein (PEBP).
**Transpor-ters**	*YHR096C*	*HXT5*	3.61	Hexose transporter with moderate affinity for glucose; induced in the presence of non-fermentable carbon sources, induced by a decrease in growth rate.
	YKL221W	*MCH2*	3.16	Protein with similarity to mammalian monocarboxylate permeases; monocarboxylate permeases are involved in transport of monocarboxylic acids across the plasma membrane.
	YER185W	*PUG1*	2.08	Plasma membrane protein involved in protoprophyrin and heme transport; roles in the uptake of protoprophyrin IX and the efflux of heme; expression is induced under both low-heme and low-oxygen conditions.
	YMR034C	*RCH1*	1.96	Putative transporter; member of the SLC10 carrier family.
	YGL104C	*VPS73*	1.75	Mitochondrial protein; mutation affects vacuolar protein sorting; putative transporter; member of the sugar porter family.
**Stress response**	*YDR034W-B*	*YDR034W-B*	3.93	Predicted tail-anchored plasma membrane protein; contains conserved CYSTM module; related proteins in other organisms may be involved in response to stress.
	YBR072W	*HSP26*	3.21	Small heat shock protein (sHSP) with chaperone activity; forms hollow, sphere-shaped oligomers that suppress unfolded proteins aggregation.
	YMR175W	*SIP18*	2.58	Phospholipid-binding hydrophilin; essential to overcome desiccation-rehydration process.
	YHR087W	*RTC3*	2.52	Protein of unknown function involved in RNA metabolism; protein abundance increases in response to DNA replication stress.
	YDL204W	*RTN2*	2.43	Reticulon protein; stabilizes membrane curvature; involved in nuclear pore assembly and maintenance of tubular ER morphology.
	YOR173W	*DCS2*	1.91	m(7)GpppX pyrophosphatase regulator; non-essential, stress induced regulatory protein.
**Others**	*YOL084W*	*PHM7*	2.81	Protein of unknown function; expression is regulated by phosphate levels.
	YMR316W	*DIA1*	2.47	Protein of unknown function; involved in invasive and pseudohyphal growth
	YMR174C	*PAI3*	2.37	Cytoplasmic proteinase A (Pep4p) inhibitor.
	YGR213C	*RTA1*	2.21	Protein involved in 7-aminocholesterol resistance; has seven potential membrane-spanning regions; expression is induced under both low-heme and low-oxygen conditions
	YPL186C	*UIP4*	2.10	Ubl (ubiquitin-like protein)-specific protease for Smt3p protein conjugates.
	YJL116C	*NCA3*	1.99	Protein involved in mitochondrion organization.
	YFR023W	*PES4*	1.92	Poly(A) binding protein, suppressor of DNA polymerase epsilon mutation.
	YLR107W	*REX3*	1.90	RNA exonuclease; required for maturation of the RNA component of RNase MRP.
	YBL049W	*MOH1*	1.89	Protein of unknown function; has homology to kinase Snf7p; not required for growth on nonfermentable carbon sources; essential for survival in stationary phase.
	YOR031W	*CRS5*	1.83	Copper-binding metallothionein.
	YDL223C	*HBT1*	1.72	Shmoo tip protein, substrate of Hub1p ubiquitin-like protein.
**Unknown**	*YMR107W*	*SPG4*	4.09	Protein of unknown function.
	YNR034W-A	*EGO4*	2.74	Protein of unknown function.

	YBR056W-A	*MNC1*	2.65	Protein of unknown function.
	YGR201C	*YGR201C*	2.48	Protein of unknown function.
	YJL016W	*TPH3*	2.35	Protein of unknown function.
	YDR391C	*YDR391C*	2.32	Protein of unknown function.
	YLR031W	*YLR031W*	2.30	Protein of unknown function.
	YMR090W	*YMR090W*	2.25	Protein of unknown function.
	YHR097C	*YHR097C*	2.23	Protein of unknown function.
	YMR118C	*SHH3*	2.13	Protein of unknown function.
	YPL247C	*YPL247C*	2.07	Protein of unknown function.
	YHR138C	*YHR138C*	2.01	Protein of unknown function.
	YNR014W	*YNR014W*	1.94	Protein of unknown function.
	YBR287W	*YBR287W*	1.91	Protein of unknown function.
	YNL195C	*YNL195C*	1.82	Protein of unknown function.
	YNL095C	*YNL095C*	1.76	Protein of unknown function.
	YDL024C	*DIA3*	1.75	Protein of unknown function.
	YLR030W	*YLR030W*	1.75	Protein of unknown function.
	YBR085C-A	*YBR085C-A*	1.73	Protein of unknown function.

Downregulated genes

GO (Function)	ORF	Gene	Ratio Akt/Akt-KD	Description
**Oxidation-reduction process**	*YAR073W*	*IMD1*	0.4	Nonfunctional protein with homology to inosine monophosphate dehydrogenase.
	YHR216W	*IMD2*	0.43	Inosine monophosphate dehydrogenase catalyzes the rate-limiting step in GTP biosynthesis. Expression is repressed by nutrient limitation.
**Cell wall organiza-tion**	*YER124C*	*DSE1*	0.52	Daughter cell-specific protein; may regulate cross-talk between the mating and filamentation pathways; deletion affects cell separation after division and sensitivity to alpha-factor and drugs affecting the cell wall.
	YNR067C	*DSE4*	0.53	Daughter cell-specific secreted protein with similarity to glucanases; degrades cell wall from the daughter side causing daughter to separate from mother.
	YNL066W	*SUN4*	0.55	Cell wall protein related to glucanases; possibly involved in cell wall septation.
	YHL028W	*WSC4*	0.59	Endoplasmic reticulum (ER) membrane protein; involved in the translocation of soluble secretory proteins and insertion of membrane proteins into the ER membrane; may also have a role in the stress response but has only partial functional overlap with WSC1-3.
	YDR528W	*HLR1*	0.60	Protein involved in regulation of cell wall composition and integrity; also involved in cell wall response to osmotic stress.

**Meiotic cell cycle**	*YML052W*	*SUR7*	0.48	Plasma membrane protein, component of eisosomes.
	YHR153C	*SPO16*	0.49	Meiosis-specific protein involved in synaptonemal complex assembly.
	YOR313C	*SPS4*	0.56	Protein whose expression is induced during sporulation.
	YMR001C	*CDC5*	0.58	Polo-like kinase; controls targeting and activation of Rho1p at cell division site via Rho1p guanine nucleotide exchange factors.
**Mitotic cell cycle**	*YOR315W*	*SFG1*	0.44	Nuclear protein putative transcription factor; required for growth of superficial pseudohyphae.
	YMR032W	*HOF1*	0.50	Protein that regulates actin cytoskeleton organization; required for cytokinesis, actin cable organization. and secretory vesicle trafficking; localized to bud neck.
	YJL194W	*CDC6*	0.51	Essential ATP-binding protein required for DNA replication.
	YGR041W	*BUD9*	0.52	Protein involved in bud-site selection.
	YJR092W	*BUD4*	0.53	Anillin-like protein involved in bud-site selection; required for the axial budding pattern; localizes with septins to the bud neck in mitosis and may constitute an axial landmark for the next round of budding.
	YPR119W	*CLB2*	0.54	B-type cyclin involved in cell cycle progression; activates Cdc28p to promote the transition from G2 to M phase.
	YGL021W	*ALK1*	0.54	Protein kinase; along with its paralog. ALK2. required for proper spindle positioning and nuclear segregation following mitotic arrest. proper organization of cell polarity factors in mitosis. proper localization of formins and polarity factors. and survival in cells that activate spindle assembly checkpoint.
	YDR146C	*SWI5*	0.56	Transcription factor that recruits Mediator and Swi/Snf complexes; activates transcription of genes expressed at the M/G1 phase boundary and in G1 phase.
	YOR025W	*HST3*	0.58	Member of the Sir2 family of NAD(+)-dependent protein deacetylases; involved along with Hst4p in telomeric silencing. cell cycle progression.
**Metabolic processes**	*YBR092C*	*PHO3*	0.46	Constitutively expressed acid phosphatase similar to Pho5p.
	YJL078C	*PRY3*	0.53	Cell wall-associated protein involved in export of acetylated sterols.
	YJL079C	*PRY1*	0.54	Sterol binding protein involved in the export of acetylated sterols.
	YPR002W	*PDH1*	0.54	Putative 2-methylcitrate dehydratase; mitochondrial protein that participates in respiration.
	YJL218W	*YJL218W*	0.58	Putative acetyltransferase.
	YER170W	*ADK2*	0.59	Mitochondrial adenylate kinase; catalyzes the reversible synthesis of GTP and AMP from GDP and ADP; may serve as a back-up for synthesizing GTP or ADP depending on metabolic conditions.
**Signaling**	*YNL145W*	*MFA2*	0.50	Mating pheromone *a*-factor; made by *a* cells; interacts with alpha cells to induce cell cycle arrest and other responses leading to mating.
	YJL157C	*FAR1*	0.58	CDK inhibitor and nuclear anchor; during the cell cycle Far1p sequesters the GEF Cdc24p in the nucleus.
	YIL015W	*BAR1*	0.59	Aspartyl protease; secreted into the periplasmic space of mating type a cell; helps cells find mating partners.
**Others**	*YDL227C*	*HO*	0.33	Site-specific endonuclease.
	YDR536W	*STL1*	0.36	Glycerol proton symporter of the plasma membrane.
	YIL057C	*RGI2*	0.40	Protein of unknown function; involved in energy metabolism under respiratory conditions; expression induced under carbon limitation and repressed under high glucose.

	YJL051W	*IRC8*	0.56	Bud tip localized protein of unknown function; mRNA is targeted to the bud by a She2p dependent transport system.
	YHR023W	*MYO1*	0.57	Type II myosin heavy chain; required for wild-type cytokinesis and cell separation.
	YBL003C	*HTA2*	0.57	Histone H2A; core histone protein required for chromatin assembly and chromosome function
	YBL002W	*HTB2*	0.60	Histone H2B; core histone protein required for chromatin assembly and chromosome function.
**Unknown**	*YIL158W*	*AIM20*	0.46	Protein of unknown function.
	YLR413W	*INA1*	0.46	Protein of unknown function.
	YMR030W-A	*YMR030W-A*	0.57	Protein of unknown function.

Supplementary Figure Legends

Fig. S1. Transmission electron microscopy of *SLM1*-overexpressing yeast cells. (A) TEM of typical BY4741 yeast cells transformed with an empty vector as a control (upper leftmost panel) or the plasmid BG1805-*SLM1* (rest of images) after 5h of incubation in SR-Gal for induction. Typical multiple PM intracellular stretches (arrowheads) and a cell showing intracellular cell wall growth (rightmost panel) upon *SLM1* overexpression. In the lower panels, typical malformations associated to the septal area of PM and cell wall (zoomed area, left) (B) Exacerbation of *SLM1* overexpression-induced defects in a *pil1*Δ mutant. Characteristic cells are shown displaying multiple cell wall intracellular growth and cytoplasmic islets (arrowheads). Y06988 (BY4741 *pil1Δ::kanMX4*) strain was transformed and processed as above. Bars represent 1 μm.

Fig. S2. (A) Graphs depicting the percentage of functional categories for upregulated (left) and downregulated (right) genes from microarray results (ratio p110α-Akt1 vs p110α-Akt1(K179M) at 6h after galactose induction. The bar charts below depict frequency of each functional category in our dataset (blue) vs. the whole genome (red). Asterisks mark categories that were significantly over-represented after chi square test with Bonferroni correction (*p=0.05; **p<0.001). (B) Overlapping of induced genes in our dataset (grey) as compared to those of García *et al*.[2] (CWI; ratio 1h Congo Red vs control; red), Gasch *et al.*[3] (Diamide treatment vs. control; yellow) and Martin *et al*. (2004)[4] (TORC2; ratio *tor2-ts* vs. WT *TOR2* 37ºC; blue). Percentages shown are relative to our Akt vs. Akt(K179M) dataset (100%).

Fig. S3. Original uncropped images corresponding to the immunoblots shown in Fig. 6, as indicated. All gels are 10% polyacrylamide except the one for Fig. 6h, which is 14%. Blots for Fig. 6a and 6b were developed by chemiluminiscence detection system (ECL™; Amersham Biosciences, UK) and films were scanned. The rest were developed using an Oddissey infrared imaging system (LI-COR) and the appropriate exposure to appreciate the phenomena described (see Methods). Images obtained in the Oddissey scanner were changed from green or red-colored images to black and white, and inverted (black bands on white background) for an optimum contrast by using Adobe Photoshop CS6 (Adobe Systems Inc.). In the blots for Fig 6g, we used plasmid-encoded HA-tagged versions of wild type and T662A Ypk1. Therefore, to

detect the phosphorylated forms of these proteins, the anti-P-Ypk1 blot (upper panel) was overexposed in order to detect lower mobility (phosphorylated) bands of phospho-Ypk1-HA (arrows), which are very minor as compared to major endogenous phospho-Ypk1 and, likely, phospho-Ypk2.

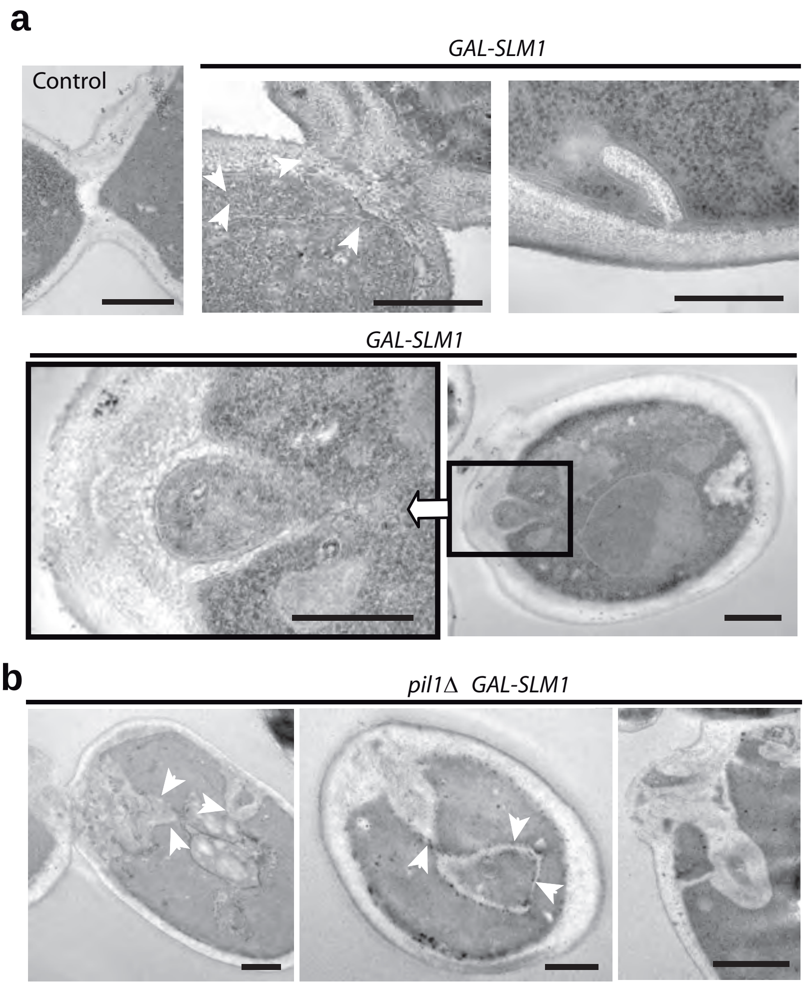

Fig. S1

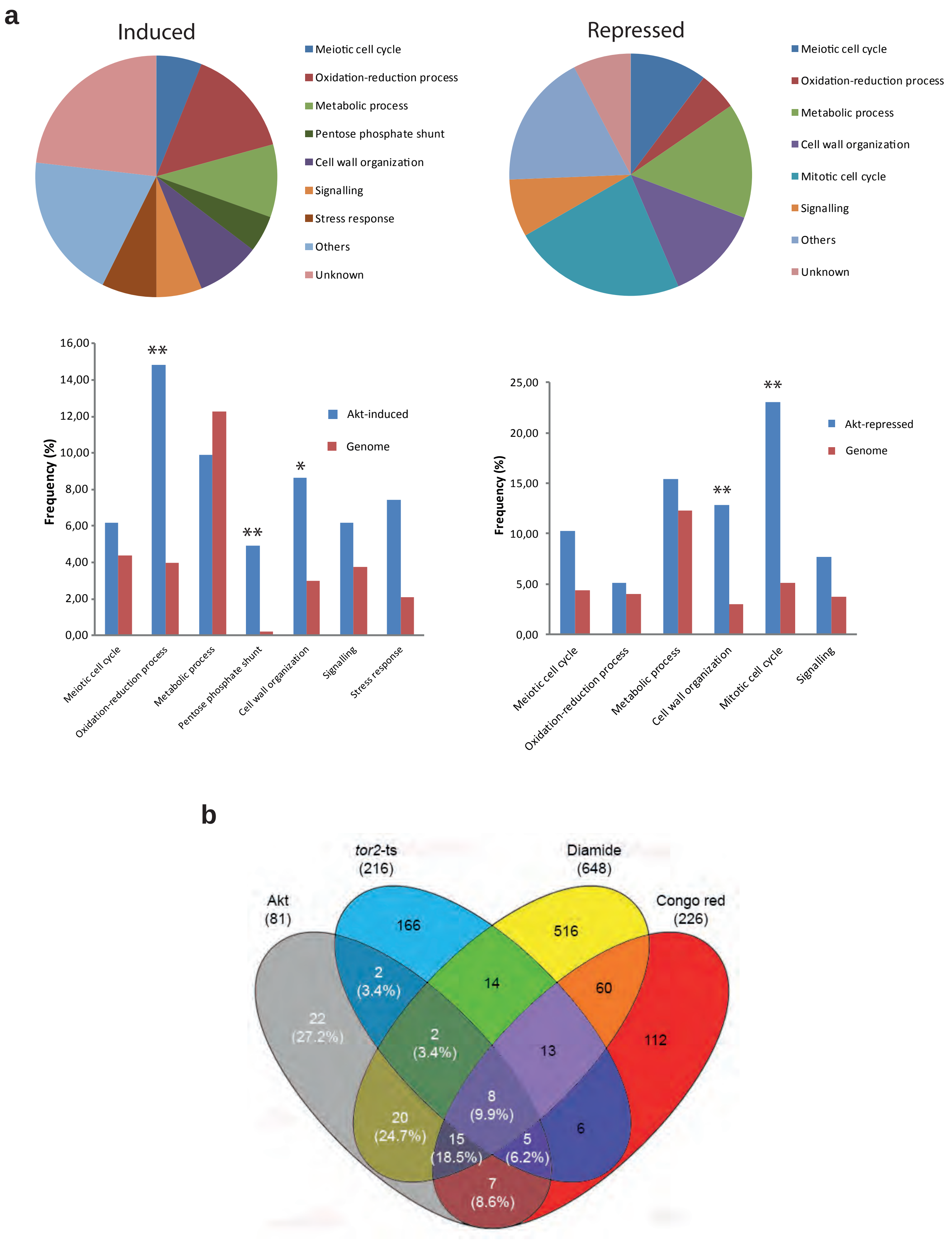
a
Induced
Meiotic cell cycle
Oxidation-reduction process
Metabolic process
Pentose phosphate shunt
Cell wall organization
Signalling
Stress response
Others
Unknown
Repressed
Meiotic cell cycle
Oxidation-reduction process
Metabolic process
Cell wall organization
Mitotic cell cycle
Signalling
Others
Unknown
Frequency (%)
16,00
14,00
12,00
10,00
8,00
6,00
4,00
2,00
0,00
**
**
*
Akt-induced
Genome
Meiotic cell cycle
Oxidation-reduction process
Metabolic process
Pentose phosphate shunt
Cell wall organization
Signalling
Stress response
Frequency (%)
25,00
20,00
15,00
10,00
5,00
0,00
**
**
Akt-repressed
Genome
Meiotic cell cycle
Oxidation-reduction process
Metabolic process
Cell wall organization
Mitotic cell cycle
Signalling
b
tor2-ts (216)
Diamide (648)
Akt (81)
Congo red (226)
166
516
2 (3.4%)
14
60
22 (27.2%)
2 (3.4%)
13
112
8 (9.9%)
20 (24.7%)
15 (18.5%)
5 (6.2%)
6
7 (8.6%)

Fig. S2

Uncropped blot for Fig. 6a

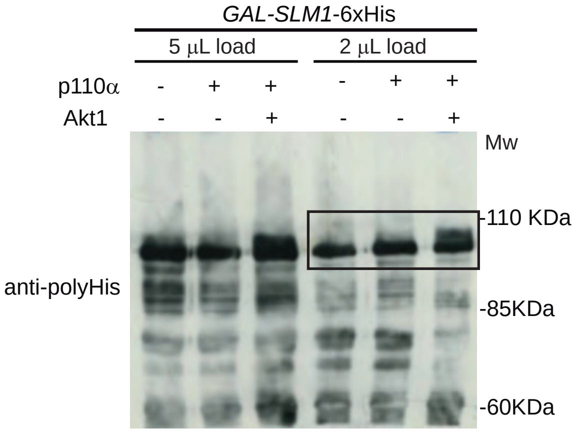

Uncropped blot for Fig. 6b

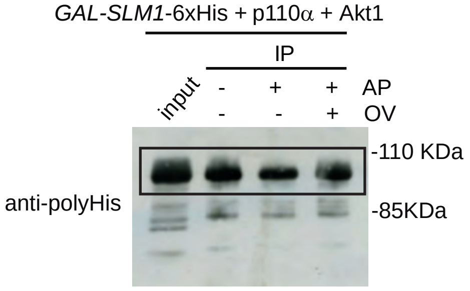

Uncropped blot for Fig. 6c

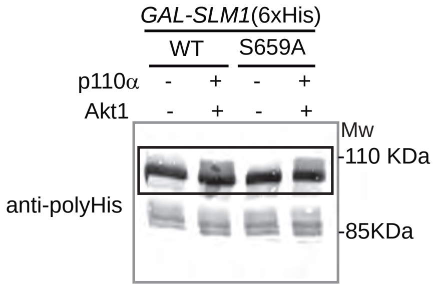

Uncropped blot for Fig. 6d

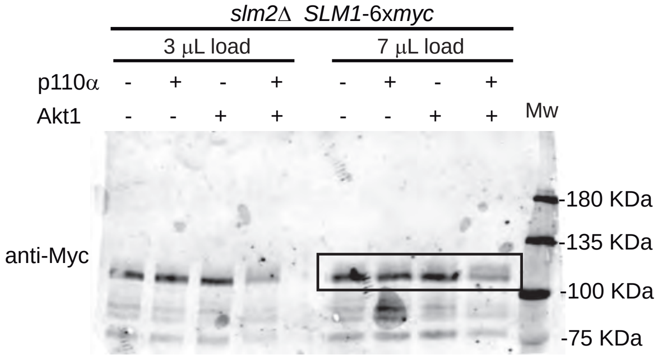

Uncropped blot for Fig. 6e

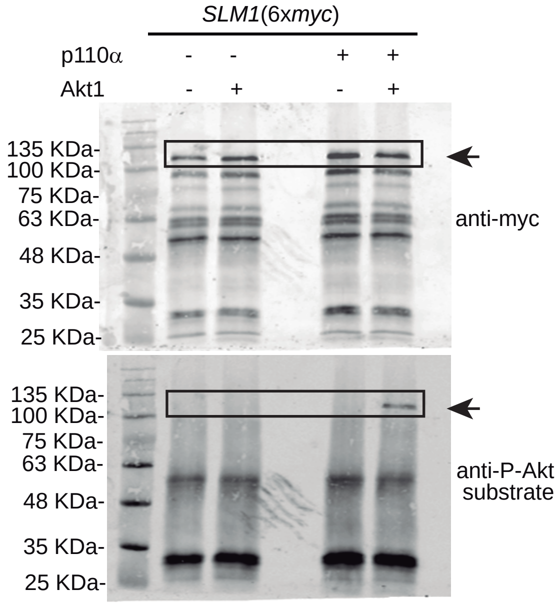

Uncropped blot for Fig. 6g

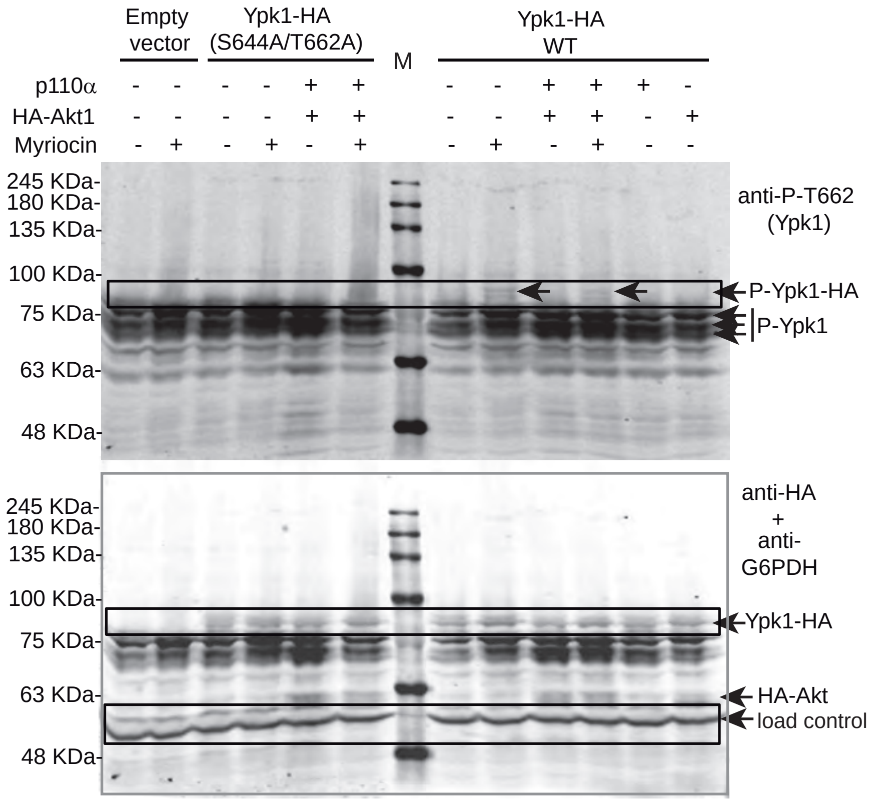

Uncropped blot for Fig. 6h

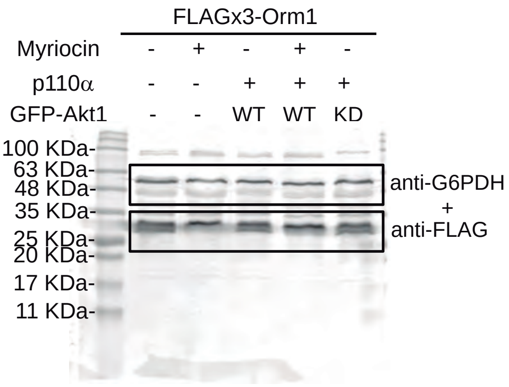

Fig. S3

Supplementary Methods

Transcriptomic analysis

RNA isolation, cDNA synthesis, microarray hybridization and image analysis were performed as described in Fernandez-Acero *et al*. (2015)[5]. Differential gene expression was expressed as a ratio obtained by dividing the average fluorescence signal of cells co-expressing p110α and Akt1 by that of those co-expressing p110α and Akt1^{K179M}, used as control. It was considered gene expression induction when the ratio was ≥1.7 and gene repression when the ratio was below ≤0.6. Statistical analysis was performed by Cyber-t program [http://cybert.microarray.ics.uci.edu][6] which calculated a Bayesian p-value in order to determine statistical significance of induction or repression data. Only data with a p-value ≤0.05 were considered for further analysis. Differentially expressed genes were functionally clustered by using bioinformatics tool Genecodis [http://genecodis.dacya.ucm.es[7]], GO Term Finder and GO Slim Mapper [SGD (http://www.yeastgenome.org)]. Statistical significance of the functional clustering and of the transcriptional factors involved in gene regulation, was determined by calculating a Bonferroni p-value, which must be below 0.05 or $1 \cdot 10^{-9}$, respectively.

References for Supplementary Material

1 Mascaraque, V. *et al.* Phosphoproteomic analysis of protein kinase C signaling in Saccharomyces cerevisiae reveals Slt2 mitogen-activated protein kinase (MAPK)-dependent phosphorylation of eisosome core components. *Mol Cell Proteomics* **12**, 557-574, doi:10.1074/mcp.M112.020438 (2013).

2 Garcia, R. *et al.* The global transcriptional response to transient cell wall damage in Saccharomyces cerevisiae and its regulation by the cell integrity signaling pathway. *J Biol Chem* **279**, 15183-15195, doi:10.1074/jbc.M312954200 (2004).

3 Gasch, A. P. *et al.* Genomic expression programs in the response of yeast cells to environmental changes. *Mol Biol Cell* **11**, 4241-4257 (2000).

4 Martin, D. E., Demougin, P., Hall, M. N. & Bellis, M. Rank Difference Analysis of Microarrays (RDAM), a novel approach to statistical analysis of microarray expression profiling data. *BMC Bioinformatics* **5**, 148, doi:10.1186/1471-2105-5-148 (2004).

5 Fernandez-Acero, T., Rodriguez-Escudero, I., Molina, M. & Cid, V. J. The yeast cell wall integrity pathway signals from recycling endosomes upon elimination of

phosphatidylinositol (4,5)-bisphosphate by mammalian phosphatidylinositol 3-kinase. *Cell Signal* **27**, 2272-2284, doi:10.1016/j.cellsig.2015.08.004 (2015).

6 Baldi, P. & Long, A. D. A Bayesian framework for the analysis of microarray expression data: regularized t -test and statistical inferences of gene changes. *Bioinformatics* **17**, 509-519 (2001).

7 Tabas-Madrid, D., Nogales-Cadenas, R. & Pascual-Montano, A. GeneCodis3: a non-redundant and modular enrichment analysis tool for functional genomics. *Nucleic Acids Res* **40**, W478-483 (2012).
